# Synergistic modulation of the gut microbiome-liver-host metabolome axis associates with the therapeutic efficacy of Danlou tablet against metabolic syndrome

**DOI:** 10.3389/fmicb.2026.1808318

**Published:** 2026-06-19

**Authors:** Minghe Yao, Xun Li, Lingling Li, Haifeng Yan, Xiaohui Li, Erping Xu, Hongru Zhou

**Affiliations:** 1 Henan University of Chinese Medicine, Zhengzhou, Henan, China; 2 Collaborative Innovation Center of Research and Development on the Whole Industry Chain of Yu-Yao, Henan Province, China; 3 The First Affiliated Hospital of Henan University of Chinese Medicine, Zhengzhou, Henan, China; 4 Collaborative Innovation Center of Prevention and Treatment of Major Diseases by Chinese and Western Medicine, Henan Province, China

**Keywords:** Danlou tablet, gut microbiome, gut-liver axis, obesity, insulin resistance, metabolomics, transcriptomics

## Abstract

**Background:**

Obesity drives chronic diseases such as cardiovascular disease and diabetes. Danlou tablet (DLT), a traditional Chinese medicine formula, is used to treat coronary heart disease by regulating lipid metabolism, suggesting potential for addressing obesity-related metabolic dysfunction. However, its role in obesity and insulin resistance remains unexplored.

**Objectives:**

We investigated the efficacy and mechanisms of DLT against high-fat diet (HFD)-induced obesity and insulin resistance.

**Methods:**

C57BL/6N mice were fed an HFD for 22 weeks and treated with DLT. A comprehensive phenotypic assessment was conducted, including body weight, glucose tolerance, insulin sensitivity, serum biochemistry, and histopathology of key tissues. To elucidate the therapeutic mechanism, we integrated 16S rRNA gene sequencing of gut microbiota, serum metabolomics (UPLC-Q-TOF-MS), and hepatic transcriptomics.

**Results:**

DLT treatment counteracted HFD-induced metabolic dysfunction, reducing body weight, adiposity, dyslipidemia, and insulin resistance, while ameliorating hepatic steatosis, inflammation, and oxidative stress. At the microbial level, DLT restored gut microbial diversity, corrected the *Firmicutes*/*Bacteroidota* ratio, and modulated key genera. Metabolomics linked these changes to restored fatty acid β-oxidation. In the liver, transcriptomics showed that DLT reversed HFD-induced gene expression, suppressed inflammatory pathways and enhanced fatty acid oxidation and xenobiotic metabolism. Integrated multi-omics analysis revealed a strong correlative relationship that DLT’s therapeutic benefits are associated with the modulation of the gut-liver axis, where remodeling of the gut microbiome is closely linked to the reprogramming of hepatic metabolic pathways.

**Conclusion:**

DLT counteracts HFD-induced obesity and insulin resistance via a multi-level regulatory mechanism that is closely associated with the modulation of the gut-liver axis, which involves suppressing pathogenic gut microbes, restoring fatty acid metabolism, and enhancing hepatic lipid catabolism and antioxidant defense. This comprehensive preclinical evidence supports the clinical translation of DLT as a novel therapeutic option for obesity and type 2 diabetes mellitus.

## Introduction

The global obesity epidemic represents a significant public health crisis, with over 70% of adults in some developed nations classified as overweight or obese (Shantaram et al., 2024). This condition is a primary driver of a cluster of metabolic disorders, including insulin resistance (IR), type 2 diabetes mellitus (T2DM), and metabolic dysfunction-associated steatotic liver disease (Cani and Van Hul, 2024). According to the International Diabetes Federation, the number of adults with diabetes reached 537 million in 2021 and is projected to rise to 783 million by 2045 (Sun et al., 2022), with T2DM accounting for over 90% of cases. The pathophysiology of these conditions is deeply rooted in IR, a state of impaired insulin signaling that leads to systemic metabolic dysregulation, including aberrant hepatic gluconeogenesis and lipolysis (Ji et al., 2025). While weight loss can lead to T2DM remission, current anti-obesity pharmacotherapies are often limited by modest efficacy and significant adverse effects, restricting their long-term clinical use (Kanbour et al., 2025; Cho et al., 2025; Liu et al., 2025; Hurtado Andrade, 2025; Kokkorakis et al., 2025; Ullah and Tamanna, 2025).

Emerging evidence has highlighted the gut microbiota as a critical regulator of host metabolism and a key player in the pathogenesis of obesity and IR (Yarahmadi et al., 2024; Miyamoto et al., 2019). The gut microbiome, often termed the body’s “second genome,” modulates energy homeostasis, inflammation, and insulin sensitivity through a complex network of microbial-derived metabolites (Ecklu-Mensah et al., 2023). Dysbiosis, an imbalance in the gut microbial community, has been consistently associated with metabolic diseases (Chong et al., 2025). This is indicative of a regulatory relationship via the gut-liver axis, a bidirectional communication pathway where microbial products, such as short-chain fatty acids, lipopolysaccharides, and bile acids, can translocate to the liver and influence hepatic metabolism and inflammation (Abdalla, 2025). Restoring microbial homeostasis and targeting the gut-liver axis therefore represent promising therapeutic strategies for metabolic disorders (Ma et al., 2025).

Traditional Chinese medicine (TCM) offers a rich source of multi-component, multi-target therapeutic agents that have been used for centuries to treat metabolic ailments (Wen et al., 2025). Danlou tablet (DLT), a modern formulation of the classic Gualouxiebai Banxia Decoction, is widely used in China for treating cardiovascular diseases such as coronary heart disease, atherosclerosis and hyperlipidemia. DLT is composed of 10 herbal medicines: *Pueraria lobata* (Willd.) Ohwi (PL), *Salvia miltiorrhiza* Bge. (SM), *Alisma orientale* (Sam.) Juzep (AO), *Astragalus membranaceus* (Fish.) Bge. var. *mongholicus* (Bge.) Hsiao (AMM), *Ligusticum chuanxiong* Hort. (LC), *Trichosanthes kirilowii* Maxim pericarp (TKM), *Drynaria fortune* (Kunze) J. Sm. (DF), *Paeonia lactiflora* Pall. (PLP), *Allium macrostemon* Bge. (AM), and *Curcuma longa* L. (CL). Previous studies have shown that its active components can modulate pathways involved in lipid synthesis and oxidative stress (Cai et al., 2024; Chen et al., 2023; Han et al., 2023; Wang et al., 2023; Xing et al., 2025; Yang et al., 2024; Zhu et al., 2024).

Furthermore, recent mechanistic studies indicate that the key active component in DLT can bind to KEAP1, inhibiting the dephosphorylation of upstream PGAM5 and downstream AIFM1. This action reduces oxidative stress-induced cell death and activates the AKT signaling pathway, which subsequently inhibits de-novo fatty acid synthesis (SREBP1-FASN-ACLY) and promotes triglyceride hydrolysis (ATGL), thereby improving hepatic lipid disorders and reducing liver fat accumulation (Zhu et al., 2025). Given DLT’s established effects on lipid metabolism and oxidative stress, it presents a compelling candidate for treating metabolic syndrome. However, the precise mechanisms through which DLT may ameliorate obesity and IR, particularly its influence on the gut microbiota and the gut-liver axis, remain unelucidated, which limits broader clinical application.

Here, we investigate the therapeutic potential of DLT in a high-fat diet (HFD)-induced mouse model of obesity and insulin resistance. We employ a multi-omics strategy, integrating 16S rRNA gene sequencing, serum metabolomics, and liver transcriptomics, to systematically unravel the mechanisms of action. We hypothesize that DLT ameliorates HFD-induced metabolic dysfunction by remodeling the gut microbiota, which in turn is closely associated with the reprogramming of host metabolism via the gut-liver axis. Our findings aim to provide a robust scientific rationale for the clinical use of DLT in the management of obesity and T2DM.

## Materials and methods

### Drugs and reagents

#### Pharmaceuticals

The study utilized Danlou tablet (DLT) and metformin as pharmaceuticals. Anhydrous glucose, ethanol, formalin, xylene, and staining kits (hematoxylin and eosin, Oil Red O, PAS) were procured from commercial suppliers for chemical and reagent purposes. Standard protocols were followed for the utilization of biochemical assay kits for total cholesterol (TC), triglycerides (TG), LDL-C, HDL-C, ALT, AST, LPS, catalase (CAT), SOD, MDA, and GSH-Px. Cytokine analysis was conducted using a multiplex immunoassay panel for leptin, insulin, resistin, IL-1α, and IL-6 (refer to Supplementary material S1).

### Animals and treatments

Fifty 6-week-old male C57BL/6N mice were obtained from Beijing Vital River Laboratory Animal Technology Co., Ltd. and housed in a clean-grade animal facility under controlled conditions (temperature: 22–24 °C, humidity: 58–60%, 12-h light/dark cycle) with free access to food and water. After a 1-week acclimation period, mice were randomly divided into five groups (*n* = 10 per group) with the following interventions:

1. Control (Con) group: Fed a standard chow diet (3.0 kcal/g; 25% protein, 60% carbohydrate, 15% fat) and administered sterile water by oral gavage daily.
2. High-fat diet (HFD) group: Fed a high-fat diet (5.24 kcal/g; 20% protein, 20% carbohydrate, 60% fat, lard as the primary fat source) and administered sterile water by oral gavage daily.
3. Metformin (MET) group: Fed the same HFD as the HFD group and administered metformin (100 mg/kg/day) by oral gavage.
4. Low-dose DLT (DLT-L) group: Fed the same HFD as the HFD group and administered DLT (680 mg/kg/day) by oral gavage.
5. High-dose DLT (DLT-H) group: Fed the same HFD as the HFD group and administered DLT (1,360 mg/kg/day) by oral gavage.

All interventions lasted for 22 weeks. During the experimental period, general health status was monitored daily, body weight was recorded weekly, and food intake was measured daily in the final week to assess caloric intake. The experimental procedures were ethically approved by the Experimental Animal Ethics Committee of Henan University of Chinese Medicine (Approval No. IACUC-202403008) and complied with national guidelines for experimental animal care. For comprehensive details on animal housing, diet formulation, and technical protocols, refer to Supplementary Methods S1.

### Glucose tolerance and insulin tolerance tests (GTT and ITT)

Glucose and insulin homeostasis were assessed through intraperitoneal glucose tolerance test (GTT) and insulin tolerance test (ITT). Blood glucose levels were monitored at baseline and at various time intervals up to 120 min post-challenge. Tolerance levels were determined by computing the area under the curve (AUC). For a comprehensive understanding of the methodologies employed, refer to the Supplementary Methods S2.

### Serum biochemistry and cytokine analysis

Blood was collected post-fasting for serum preparation. An automated chemistry analyzer measured serum lipids and liver enzymes, while ELISA and multiplex immunoassay quantified oxidative stress/inflammatory markers and metabolic hormones. HOMA-IR estimated insulin resistance. Supplementary Methods S3, provides detailed procedures, and reagent sources are listed in Supplementary material S1.

### UHPLC-Q-orbitrap HRMS analysis

The separation of samples was performed on a Dionex^™^ UltiMate^™^ 3000 UPLC platform. Data acquisition was conducted in “Full scan/dd-MS^2^” mode. For the full scan, parameters were: 70,000 resolution, 1 × 10^6^ AGC target, 50 ms maximum isolation time, and a scan range covering *m*/*z* 100–1,500. Regarding the dd-MS^2^ scans, the resolution was 17,500 with an AGC target of 1 × 10^5^ and a 50 ms injection time. The top *n* (*n* ≤ 10) most intense precursors were fragmented using a dynamic exclusion setting, a 2 *m*/*z* isolation window, collision energies of 10, 30, and 60 V, and an intensity threshold of 1 × 10^5^. Progenesis QI software (version 3.0, Waters Corp., Milford, MA, United States) was used to process the data, encompassing import, peak extraction, and deconvolution. Compound identification involved screening the TCM Pro 2.0 reference database (Beijing Hexin Technology Co., Ltd.) alongside a theoretical database constructed from literature and public data. Reliable identification was established based on retention time and mass errors, daughter ion matching degrees, isotope distribution, and peak area.

### Tissue collection and histology

Mice were anesthetized with an intraperitoneal injection of 3% pentobarbital sodium (45 mg/kg) to ensure deep anesthesia, which was verified by the loss of righting reflex and absence of pain response to paw pinch. Euthanasia was subsequently performed via cervical dislocation, a method compliant with the American Veterinary Medical Association (AVMA) Guidelines for the Euthanasia of Animals.

Subsequently, major metabolic tissues (liver, subcutaneous adipose tissue, epididymal adipose tissue, etc.) were rapidly excised and weighed to determine organ indices. Liver and adipose tissues underwent standard histological staining: hematoxylin and eosin (H&E) staining for morphological observation, Oil Red O staining (for hepatic lipid accumulation), and periodic acid–Schiff (PAS) staining (for hepatic glycogen detection). All animal procedures, including blood collection, anesthesia, and euthanasia, were approved by the Experimental Animal Ethics Committee of Henan University of Chinese Medicine (Approval No. IACUC-202403008) and strictly complied with national guidelines for the care and use of laboratory animals, as well as international ethical standards for animal experimentation. For comprehensive details on staining procedures and technical parameters, refer to Supplementary Methods S4.

### 16S rRNA gene sequencing and microbiome analysis

Fecal samples collected at week 20 underwent V3–V4 16S rRNA gene amplicon sequencing on an Illumina paired-end platform. The reads underwent processing in QIIME2 with DADA2 to produce ASVs and were taxonomically classified against a curated reference database. Standard workflows were employed to analyze diversity metrics, differential taxa, and functional inference. For a comprehensive understanding of the methodologies and parameters utilized, refer to Supplementary Methods S5.

### Untargeted serum metabolomics

Serum metabolomics analysis was conducted through untargeted UHPLC-Q-TOF-MS in positive and negative ion modes. The data underwent processing via an XCMS-based workflow and annotation through MS/MS matching with public databases. For comprehensive details on sample preparation, LC-MS configurations, and data processing parameters, please refer to Supplementary Methods S6.

### Liver transcriptomics

Liver tissue total RNA was extracted and sequenced using an Illumina platform in a paired-end manner. The resulting clean reads were then aligned to the mouse reference genome (GRCm39) for expression quantification. Differential expression genes (DEGs) were determined utilizing DESeq2 with multiple-testing correction. Subsequently, functional enrichment and network analyses were conducted. Specific quality control thresholds and pipeline parameters can be found in Supplementary Methods S7.

### Statistical analysis

Data are reported as mean ± SEM. Group comparisons and longitudinal analyses utilized ANOVA-based frameworks with suitable *post-hoc* corrections, where *p* < 0.05 indicated statistical significance. Additional statistical information can be found in Supplementary Methods S8.

## Results

### Chemical components analysis of DLT

**Figure 1:**
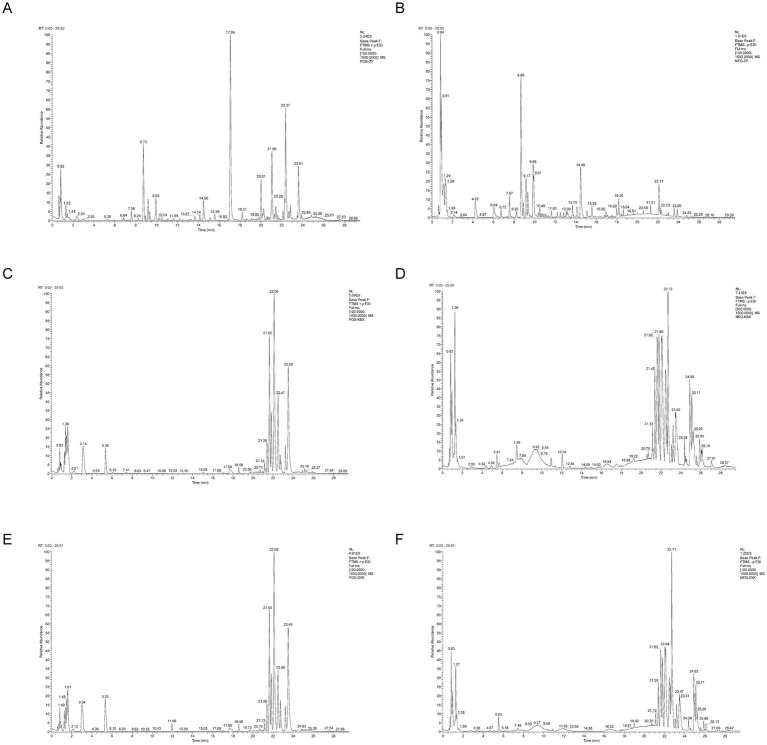
The main components of DLT against obesity and insulin resistance in mice. **(A)** Base peak ions (BPI) chromatogram of DLT detected in positive mode. **(B)** Base peak ions (BPI) chromatogram of DLT detected in negative mode. **(C)** Base peak ions (BPI) chromatogram of blank serum detected in positive mode. **(D)** Base peak ions (BPI) chromatogram of blank serum detected in negative mode. **(E)** Base peak ions (BPI) chromatogram of DLT-containing serum detected in positive mode. **(F)** Base peak ions (BPI) chromatogram of DLT-containing serum detected in negative mode.

We utilized UHPLC-Q-Orbitrap HRMS to comprehensively analyze the chemical composition of DLT, aiming to preliminarily characterize the chemical profile of the formula and screen potential candidate components underlying its efficacy in treating obesity and insulin resistance. Figures 1A,B present the base peak chromatograms (BPCs) obtained under positive and negative ion modes. A total of 171 components were characterized in DLT by matching their retention times, parent ion mass errors, fragment ions, and isotope distributions against reference standards and literature records. The identified compounds were mainly classified as prenol lipids, isoflavonoids, organooxygen compounds, flavonoids, steroids and steroid derivatives, etc. All measurements showed a mass error within ±5 ppm, and detailed data are provided in Supplementary Table S1.

Given that DLT exerts its therapeutic effects following digestion and absorption into the bloodstream, we further investigated the serum chemistry of DLT-treated and blank group rats (Figures 1C,D). We detected 33 absorbed components in the serum of the DLT group, including 27 prototypes and 6 metabolites. The corresponding BPCs and identification findings are shown in Figures 1E,F and Supplementary Table S2. These blood-entering components may serve as potential candidate active constituents involved in DLT’s regulation of obesity and insulin resistance.

### DLT attenuates high-fat diet-induced obesity and adiposity

**Figure 2:**
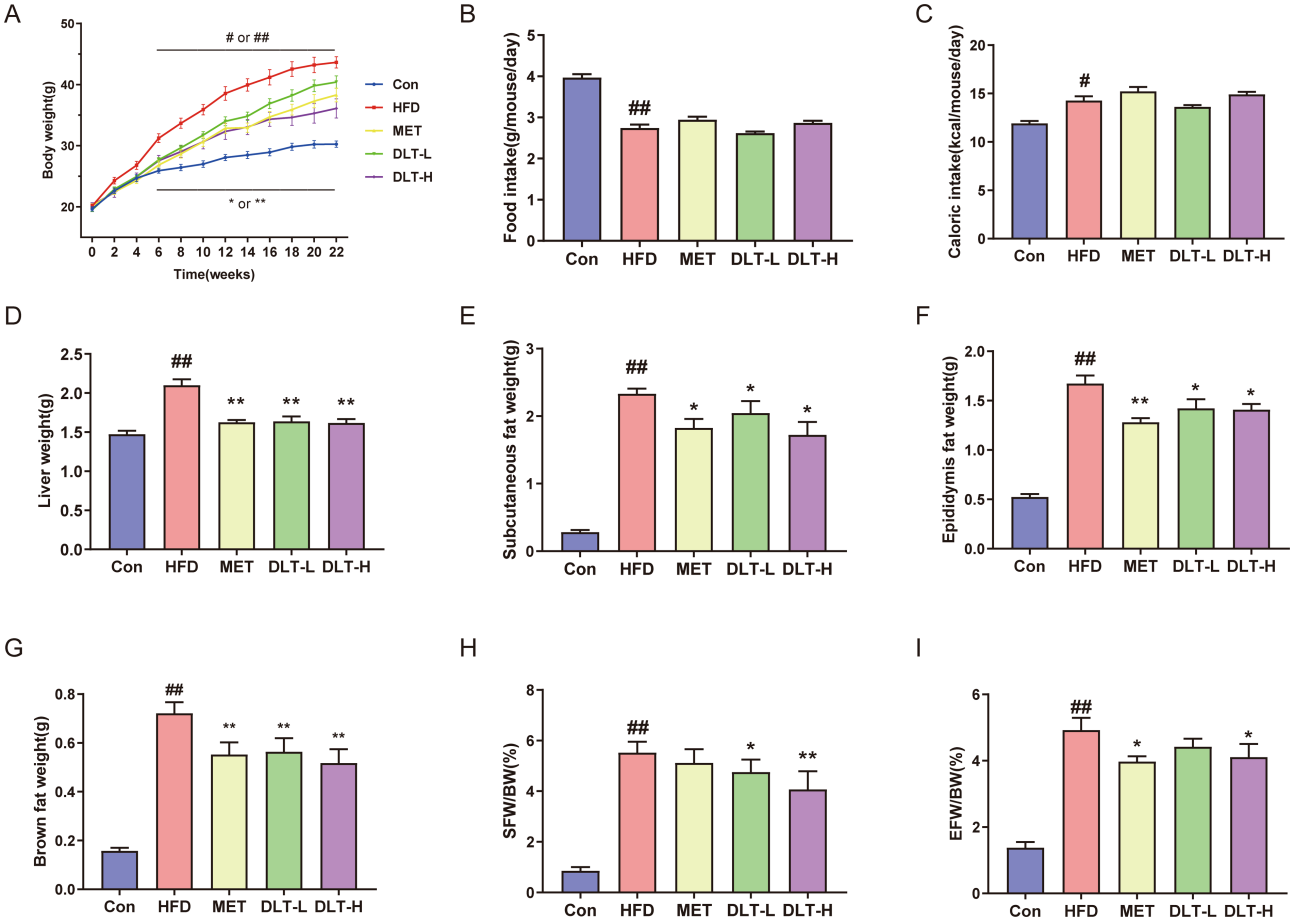
DLT treatment ameliorates high-fat diet (HFD)-induced obesity and adiposity in mice. **(A)** Time course of body weight changes over the 22-week study (*n* = 10). **(B)** Average daily food intake (g/mouse/day). **(C)** Average daily caloric intake (kcal/mouse/day). **(D)** Weight of subcutaneous fat. **(E)** Weight of epididymal fat. **(F)** Liver weight. **(G)** Weight of brown fat. **(H)** Ratio of epididymal fat weight to body weight (SFW/BW, %). **(I)** Ratio of epididymal fat weight to body weight (EFW/BW, %). For panels **(B–H)**, data are presented as mean ± SEM (*n* = 10 per group). Statistical significance was determined by one-way ANOVA with Dunnett’s *post-hoc* test. ^##^*p* < 0.01 vs. Con group; ^*^p < 0.05, ^**^p < 0.01 vs. HFD group.

To validate the anti-obesity potential of DLT observed in preliminary studies (Chen et al., 2023), we first assessed its impact on body weight and adiposity. We monitored body weight over a 22-week period in mice fed a high-fat diet (HFD). While the HFD group exhibited significantly higher body weights compared to the chow-fed control (Con) group from week 6 onward, treatment with both low (DLT-L) and high (DLT-H) dose DLT suppressed this HFD-induced weight gain (Figure 2A). Despite gaining more weight, the HFD group and all treatment groups consumed less food by weight compared to the Con group. However, there was no significant difference in food intake between the HFD, MET, DLT-L, and DLT-H groups (Figure 2B). When the caloric density of the diets was considered, the daily caloric intake for the HFD and treatment groups was significantly higher than that of the Con group, yet there was no significant difference among the HFD-fed groups (Figure 2C). This demonstrates that the therapeutic effect of DLT occurred without affecting the volume of food consumed relative to other HFD-fed mice. These findings confirm that DLT mitigates HFD-induced weight gain through mechanisms independent of reduced caloric intake.

The weight-reducing effects of DLT were confirmed by significant decreases in adiposity at the study’s endpoint. HFD feeding led to a marked accumulation of brown, subcutaneous, and epididymal fat, as well as an increase in liver weight (Figures 2D–G). Treatment with DLT-L, DLT-H, and the standard drug MET significantly reduced the absolute weights of brown, subcutaneous, and epididymal fat compared to the HFD group. This effect was also evident in the DLT and MET treatments for liver weight. To account for differences in final body weight, we calculated tissue-to-body-weight ratios. Both DLT-L and DLT-H significantly reduced the ratio of subcutaneous fat to body weight compared to the HFD group, with the high dose having the most pronounced effect (Figure 2H). Similarly, DLT-H significantly lowered the epididymal fat-to-body-weight ratio (Figure 2I). These endpoint data demonstrate that DLT effectively counteracts HFD-induced fat accumulation in multiple depots, solidifying its role as an anti-obesity agent.

### DLT restores glucose homeostasis and insulin sensitivity in HFD-fed mice

**Figure 3:**
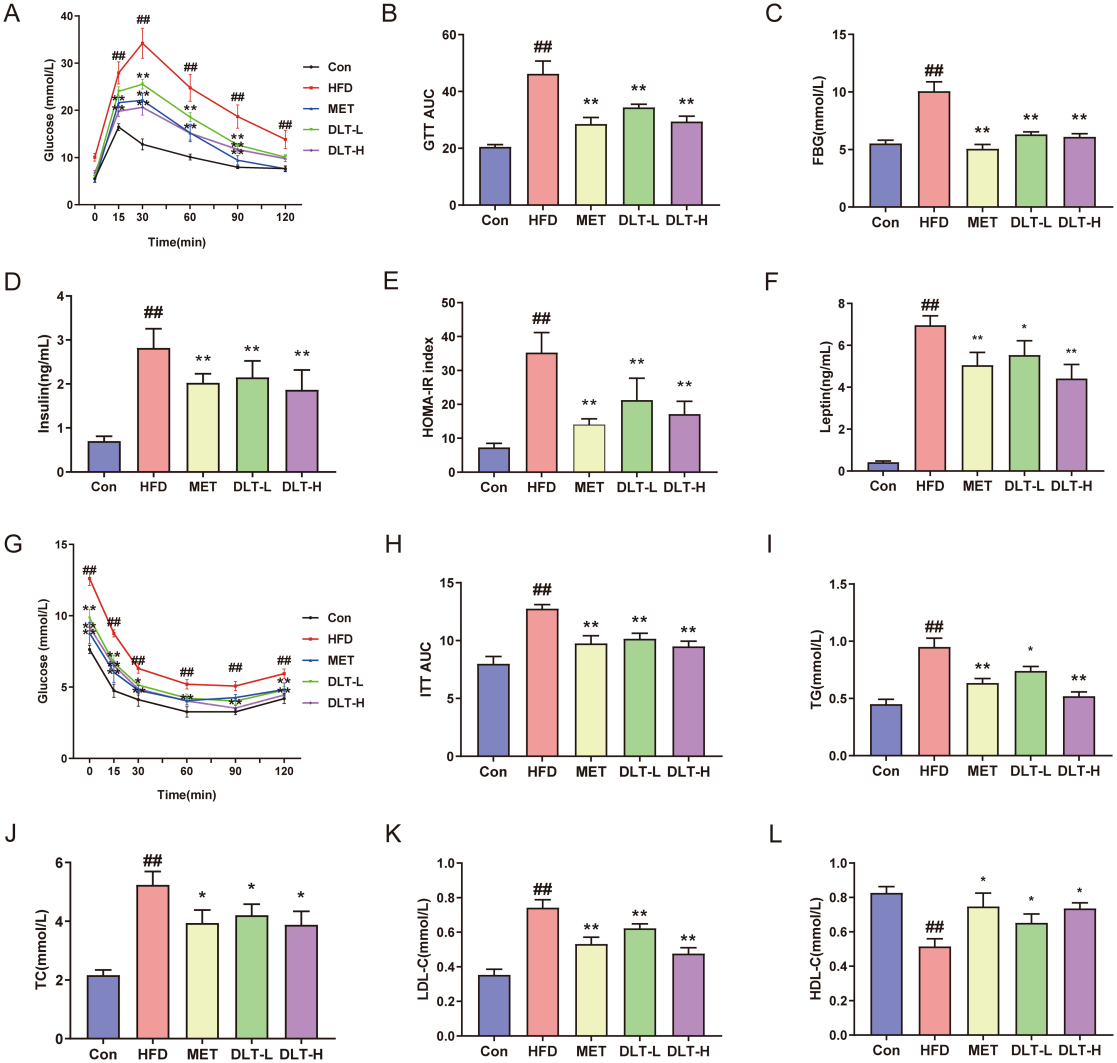
DLT treatment improves glucose homeostasis, insulin sensitivity, and the serum lipid profile in HFD-fed mice. **(A)** Intraperitoneal glucose tolerance test (GTT) curves. **(B)** Area under the curve (AUC) for the GTT. **(C)** Fasting blood glucose (FBG) levels. **(D)** Serum insulin levels. **(E)** Homeostasis model assessment of insulin resistance (HOMA-IR) index. **(F)** Serum leptin levels. **(G)** Intraperitoneal insulin tolerance test (ITT) curves. **(H)** Area under the curve (AUC) for the ITT. **(I)** Serum triglyceride (TG) levels. **(J)** Serum total cholesterol (TC) levels. **(K)** Serum low-density lipoprotein cholesterol (LDL-C) levels. **(L)** Serum high-density lipoprotein cholesterol (HDL-C) levels. For line graphs **(A,G)**, data points represent mean values, and significance (## or **) indicates a difference from the Con or HFD group, respectively, at that specific time point. For bar graphs **(B–F,H–L)**, data are presented as mean ± SEM (*n* = 10 per group). Statistical significance was determined by two-way ANOVA with repeated measures for **(A,G)**, and one-way ANOVA with Dunnett’s *post-hoc* test for the bar graphs. ^#^*p* < 0.05, ^##^*p* < 0.01 vs. Con group; ^*^p < 0.05, ^**^p < 0.01 vs. HFD group.

Given the well-established link between obesity and metabolic dysfunction (Yang et al., 2022), we next investigated the effects of DLT on glucose homeostasis and insulin sensitivity. To assess the therapeutic potential of DLT on glucose homeostasis, we performed glucose and insulin tolerance tests. In the intraperitoneal glucose tolerance test (GTT), the HFD group showed significantly impaired glucose tolerance compared to the Con group, with elevated blood glucose at all measured time points and a significantly larger area under the curve (AUC) (Figures 3A,B). Treatment with DLT-L, DLT-H, and MET all significantly improved glucose clearance, resulting in significantly lower GTT AUCs compared to the HFD group (Figure 3B). These results indicate that DLT is highly effective at improving glucose handling, performing on par with the standard drug, metformin.

Consistent with these findings, all three treatments also improved insulin sensitivity. Fasting blood glucose (FBG) was significantly reduced in the MET, DLT-L, and DLT-H groups compared to the HFD group, despite the MET group being slightly lower than the Con group (Figure 3C). Similarly, serum insulin levels and the HOMA-IR index, which were significantly elevated in the HFD group, were all significantly reduced by the treatments (Figures 3D,E). The insulin tolerance test (ITT) confirmed these results; the HFD group displayed significant insulin resistance, while the MET, DLT-L, and DLT-H groups all demonstrated significantly improved insulin sensitivity (Figures 3G,H). In contrast, while HFD feeding led to a significant increase in serum leptin, treatment with MET, DLT-L, or DLT-H did not result in a statistically significant change in leptin levels (Figure 3F). Taken together, these data demonstrate that DLT treatment effectively restores insulin sensitivity, though this benefit does not appear to be mediated through changes in circulating leptin levels.

We next evaluated the effects of DLT on the lipid profile. HFD feeding induced severe dyslipidemia, characterized by elevated triglycerides (TG), total cholesterol (TC), and LDL-C, alongside reduced HDL-C (Figures 3I–L). DLT-H treatment significantly reduced serum TG levels, an effect comparable to MET. For TC and LDL-C, both DLT-L and DLT-H treatment induced a significant reduction, while MET had a less pronounced but still significant effect (Figures 3J,K). Importantly, the reduction in TC and LDL-C was more significant with DLT-H than with MET. Finally, the HFD-induced decrease in HDL-C was significantly ameliorated by DLT-L and DLT-H treatment, while MET’s effect did not reach significance (Figure 3L). Collectively, these data confirm that DLT corrects the dyslipidemic profile caused by a high-fat diet, with the high dose showing superior efficacy to metformin for key lipid parameters.

### DLT alleviates HFD-induced inflammatory response, oxidative stress, and hepatic steatosis in mice

**Figure 4:**
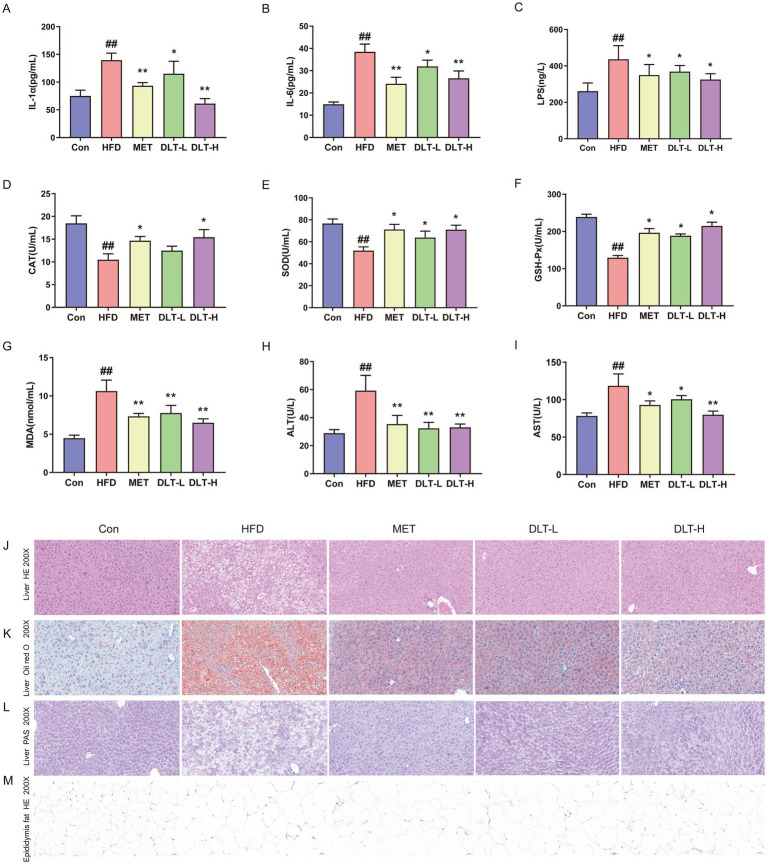
DLT treatment ameliorates HFD-induced systemic inflammation, oxidative stress, and hepatic steatosis. **(A)** Serum interleukin-1α (IL-1α) level. **(B)** Serum interleukin-6 (IL-6) level. **(C)** Serum lipopolysaccharide (LPS) level. **(D)** Serum catalase (CAT) concentration. **(E)** Serum superoxide dismutase (SOD) level. **(F)** Serum glutathione peroxidase (GSH-Px) content. **(G)** Serum malondialdehyde (MDA) content. **(H)** Serum alanine aminotransferase (ALT) level. **(I)** Serum aspartate aminotransferase (AST) level. **(J)** Representative images of hematoxylin and eosin (H&E) staining of liver sections, showing hepatocyte morphology and lipid droplet accumulation. **(K)** Representative images of Oil Red O staining of liver sections, visualizing triglyceride deposition (red). **(L)** Representative images of periodic acid–Schiff (PAS) staining of liver sections, indicating glycogen content (magenta). **(M)** Representative images of H&E staining of epididymal adipose tissue, showing adipocyte size. Scale bars represent 200 μm. For all bar graphs **(A–I)**, data are presented as mean ± SEM (*n* = 10 per group). Statistical significance was determined by one-way ANOVA with Dunnett’s *post-hoc* test. ^#^*p* < 0.05, ^##^*p* < 0.01 vs. Con group; ^*^p < 0.05, ^**^p < 0.01 vs. HFD group.

Chronic metabolic overload from a high-fat diet is known to trigger systemic inflammation, oxidative stress, and hepatic injury (Duan et al., 2018). We therefore assessed these critical pathological hallmarks. Chronic HFD feeding induced a significant inflammatory response, as shown by elevated serum levels of the pro-inflammatory cytokines IL-1α and IL-6, and the bacterial endotoxin LPS (Figures 4A–C). While MET did not significantly change IL-1α or LPS, treatment with DLT, particularly at the high dose (DLT-H), led to a significant reduction in all three inflammatory mediators. These results demonstrate the superior anti-inflammatory efficacy of DLT-H in mitigating HFD-induced systemic inflammation.

The HFD also caused significant oxidative stress, marked by a decrease in antioxidant enzymes (CAT, SOD, GSH-Px) and an increase in the lipid peroxidation marker MDA (Figures 4D–G). DLT treatment effectively counteracted these effects in a dose-dependent manner. DLT-H significantly restored the activities of all three antioxidant enzymes and significantly reduced MDA levels, performing as well as or better than MET. Collectively, these findings confirm that DLT possesses potent antioxidant properties that effectively restore the redox balance disrupted by a high-fat diet.

Furthermore, HFD-induced metabolic stress led to liver damage, as indicated by significantly elevated serum levels of the liver injury markers ALT and AST (Figures 4H,I). Both DLT-L and DLT-H, as well as MET, significantly reduced these marker levels back towards the control, indicating a hepatoprotective effect. These biochemical results strongly suggest that DLT can protect the liver from the functional damage caused by metabolic stress.

To verify these biochemical findings at the tissue level, we performed a histological analysis. Histological analysis confirmed these biochemical findings. H&E staining of liver tissue revealed that HFD caused pronounced hepatocyte swelling and the accumulation of large lipid droplets, while the control group showed intact, well-organized hepatocytes (Figure 4J). DLT treatment effectively ameliorated these morphological abnormalities, resulting in a liver structure much closer to that of the control. Oil Red O staining, which specifically visualizes neutral lipids, confirmed severe triglyceride deposition in the livers of HFD-fed mice, evidenced by intense red staining (Figure 4K). This pathological lipid accumulation was significantly attenuated by DLT administration, with staining patterns resembling the control group. Additionally, PAS staining, which highlights glycogen content, demonstrated that the HFD group had a marked depletion of hepatic glycogen, which was effectively restored by both doses of DLT (Figure 4L), suggesting improved hepatic glucose storage. In the epididymal adipose tissue, HFD feeding induced significant adipocyte hypertrophy, which was effectively prevented by DLT treatment (Figure 4M). In summary, the histological evidence demonstrates that DLT provides robust protection against HFD-induced hepatic steatosis, restores healthy liver glycogen storage, and prevents pathological adipose tissue expansion.

### DLT reverses gut dysbiosis in HFD-fed mice

**Figure 5:**
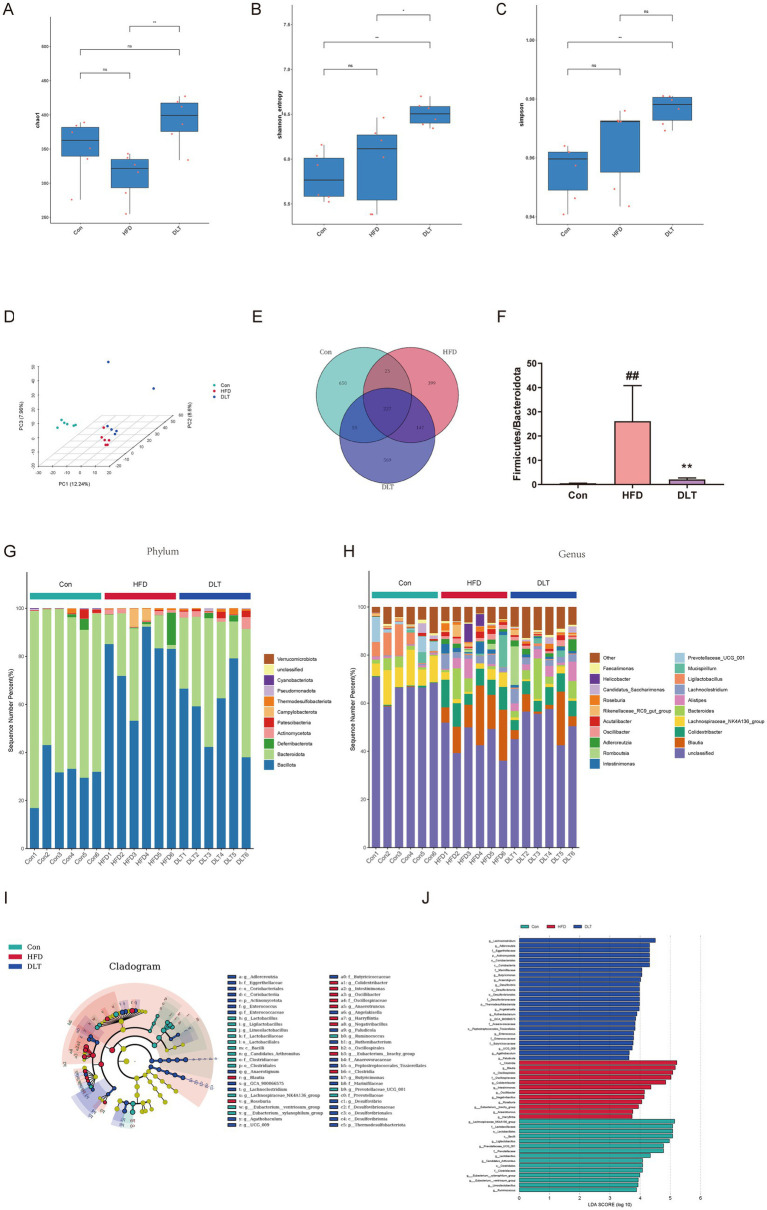
DLT treatment modulates the gut microbiota composition and diversity in HFD-fed mice. **(A–C)** Alpha diversity metrics of the gut microbiota, including Chao1 richness **(A)**, Shannon diversity index **(B)**, and Simpson evenness index **(C)**. **(D)** Three-dimensional principal coordinates analysis (PCoA) plot showing the beta diversity of gut microbial communities, based on Bray–Curtis distance. **(E)** Venn diagram illustrating the number of shared and unique operational taxonomic units (OTUs) among the Control (Con), high-fat diet (HFD), and DLT groups. **(F)** The ratio of Firmicutes to Bacteroidota (F/B) phyla. **(G)** Stacked bar chart showing the relative abundance of gut microbial communities at the phylum level. **(H)** Stacked bar chart showing the relative abundance of gut microbial communities at the genus level. **(I)** Cladogram from linear discriminant analysis effect size (LEfSe) analysis, showing the phylogenetic distribution of differentially abundant taxa among the three groups. The central point represents the kingdom, and each ring represents a subsequent taxonomic level (phylum to genus). **(J)** Histogram of the linear discriminant analysis (LDA) scores for differentially abundant genera, with an LDA score threshold of >3.0. The length of the bar represents the effect size, and the color indicates the group in which the taxon is most abundant. For box plots **(A–C,F)**, data are presented as mean ± SD (*n* = 10). Statistical significance was determined by Kruskal–Wallis test with Dunn’s *post-hoc* test. ^#^*p* < 0.05, ^##^*p* < 0.01 vs. Con group; ^*^p < 0.05, ^**^p < 0.01 vs. HFD group.

It is well-established that obesity and high-fat diets are major disruptors of gut microbiota homeostasis (Wang et al., 2023). Given that the high dose of DLT (1,360 mg/kg) demonstrated superior efficacy, we selected it for subsequent 16S rRNA gene sequencing to characterize its impact on the gut microbiota. Alpha diversity analysis, which measures the richness and evenness within each sample, revealed that a HFD trended towards a reduction in the Chao1, Shannon, and Simpson indices compared to the Control group, though this did not reach statistical significance (Figures 5A–C). This suggests that HFD initiated a loss of microbial diversity, which is a hallmark of dysbiosis. Treatment with DLT significantly increased the Shannon and Simpson indices compared to both the Con and HFD groups, indicating that DLT effectively restored and even enhanced gut microbial diversity (Figures 5B,C).

Beyond simple diversity metrics, understanding the differences in overall community structure (beta diversity) is critical. To assess this, we performed beta diversity analysis. A 3D principal coordinates analysis (PCoA) plot revealed clear and distinct clustering of samples from the three groups (Figure 5D). The Venn diagram shows the shared abundance of gut microbiota, where 227 OTUs were common among the Con, HFD, and DLT groups, while the Con, HFD, and DLT groups had 650, 399, and 569 unique OTUs, respectively (Figure 5E). This demonstrates that HFD feeding caused a major shift in the overall gut microbial structure, which was significantly altered by DLT treatment, creating a microbial profile distinct from both HFD and control mice.

A key feature of diet-induced dysbiosis is the alteration of major phyla and the overall balance of the community (Brown et al., 2012). At a compositional level, the HFD-fed mice displayed a significant increase in the Firmicutes/Bacteroidota (F/B) ratio, a well-established marker of gut dysbiosis (Magne et al., 2020). DLT treatment effectively reversed this shift, bringing the F/B ratio back towards a healthier state (Figure 5F). At the phylum level, the high dose of DLT counteracted HFD-induced alterations by promoting the recovery of the beneficial Bacteroidota phylum and suppressing the abnormal expansion of Deferribacterota (Figure 5G). At the genus level, this beneficial effect was even more pronounced. DLT treatment notably inhibited the overgrowth of inflammation-associated genera such as *Mucispirillum* and restored the abundance of genera that have been widely reported in previous studies to be associated with short-chain fatty acid (SCFA) production, including *Bacteroides* and *Blautia* (Figure 5H). These data confirm that DLT exerts a powerful normalizing effect on the composition of the gut microbiota at multiple taxonomic levels based on 16S rRNA sequencing at the genus level.

While broad taxonomic changes are important, identifying the specific bacterial taxa driving these differences provides deeper insight. To pinpoint these key players, we employed LEfSe analysis. The cladogram from this analysis revealed that the gut microbiota of DLT-treated mice was significantly enriched for lactic acid bacteria under the phylum Bacillota, including *Lactobacillus* and *Ligilactobacillus*. In stark contrast, the HFD group was characterized by an enrichment of potentially pathogenic bacteria, such as *Desulfovibrio* (Figure 5I). Linear discriminant analysis (LDA) further quantified these differences, showing that DLT treatment was strongly associated with beneficial microbial genera reported to exert metabolic benefits, like *Lactobacillales*, *Lactobacillus*, and the butyrate-producing family Butyricicoccaceae, while the HFD group was dominated by harmful bacteria from the Desulfovibrionales order (Figure 5J). In summary, these detailed bioinformatic analyses demonstrate that DLT effectively counteracts HFD-induced gut microbiota dysbiosis by selectively suppressing pathogen-associated genera and promoting the proliferation of microbial genera with reported beneficial metabolic and immunomodulatory properties.

### DLT modulates HFD-induced alterations in key microbial genera

**Figure 6:**
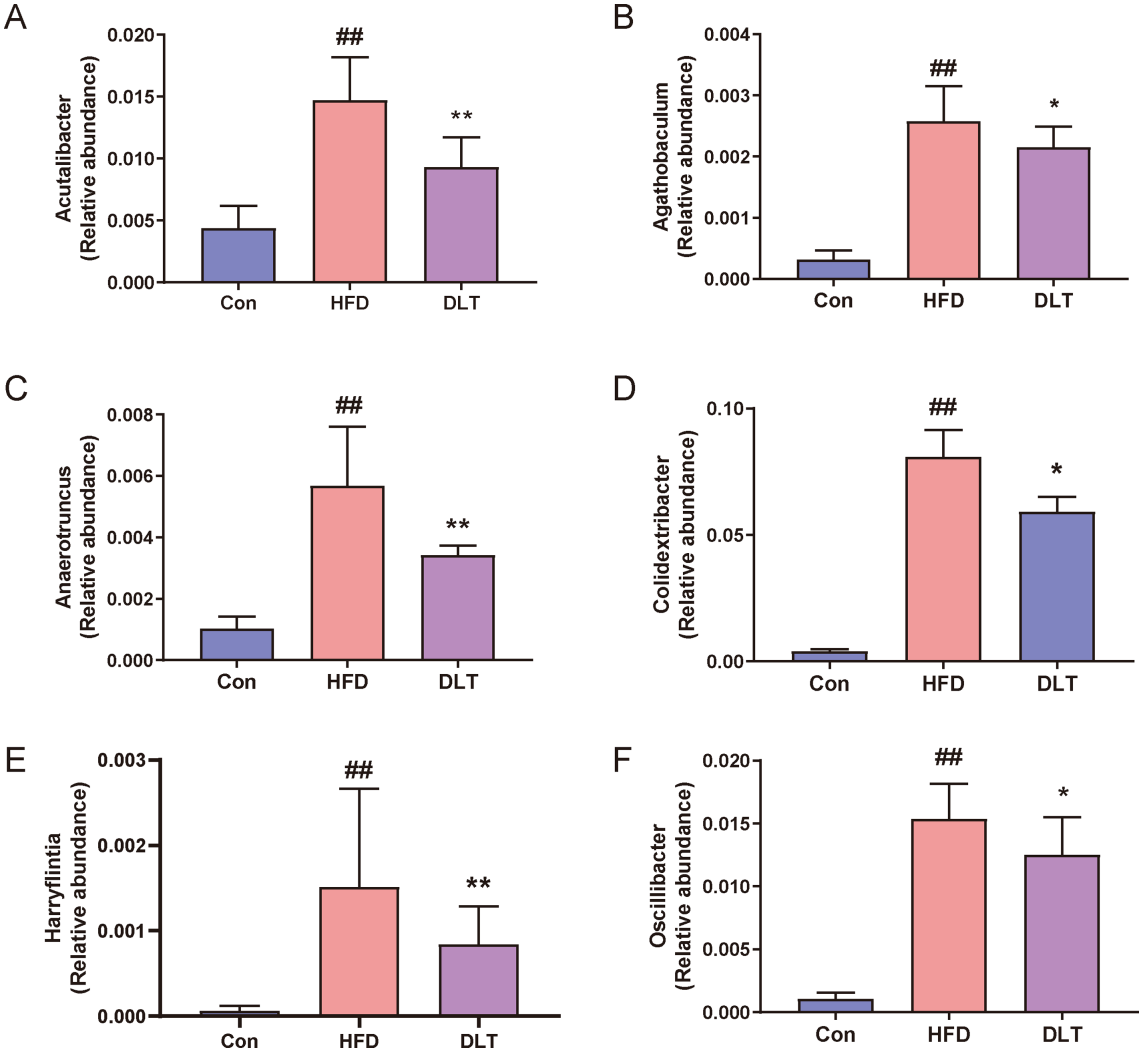
DLT treatment normalizes the relative abundance of key gut microbiota at the genus level. **(A)** Relative abundance of Acutalibacter. **(B)** Relative abundance of Agathobaculum. **(C)** Relative abundance of Anaerotruncus. **(D)** Relative abundance of Colidextribacter. **(E)** Relative abundance of Harryflintia. **(F)** Relative abundance of Oscillibacter. For all bar graphs, data are expressed as the mean ± SEM (*n* = 6 per group). Statistical significance was determined by one-way ANOVA with Dunnett’s post-hoc test. ^##^*p* < 0.01 vs. the Con group; ^*^p < 0.05, ^**^p < 0.01 vs. the HFD group.

To further elucidate the specific bacterial genera targeted by DLT, we performed a focused analysis on the relative abundance of key taxa identified from our broader sequencing data. Consistent with the HFD-induced gut dysbiosis, the high-fat diet led to a significant increase in the relative abundance of multiple bacterial genera compared to the control group. This was observed for microbial genera that are commonly associated with SCFA production in previous studies such as *Blautia* and *Roseburia*, as well as other HFD-responsive genera including *Agathobaculum*, *Colidextribacter*, *Oscillibacter*, *Acutalibacter*, *Anaerotruncus*, *Harryflintia*, and [Eubacterium]_brachy_group (Figures 6A–F). These results confirm that HFD profoundly disrupts the abundance of key genera, even those typically reported as beneficial in metabolic regulation in existing literature. Crucially, DLT intervention effectively reversed the HFD-induced upregulation of these same genera. For all nine genera examined, DLT treatment significantly reduced their abundance back down to levels that were not significantly different from the control group (Figures 6A–I). This targeted suppression by DLT indicates a powerful regulatory effect, normalizing the dysbiotic expansion of specific bacterial populations caused by the high-fat diet. This finding provides key evidence for the mechanism by which DLT restores overall gut microbial balance at the genus-level taxonomic resolution.

### DLT attenuates HFD-induced metabolic disorders

**Figure 7:**
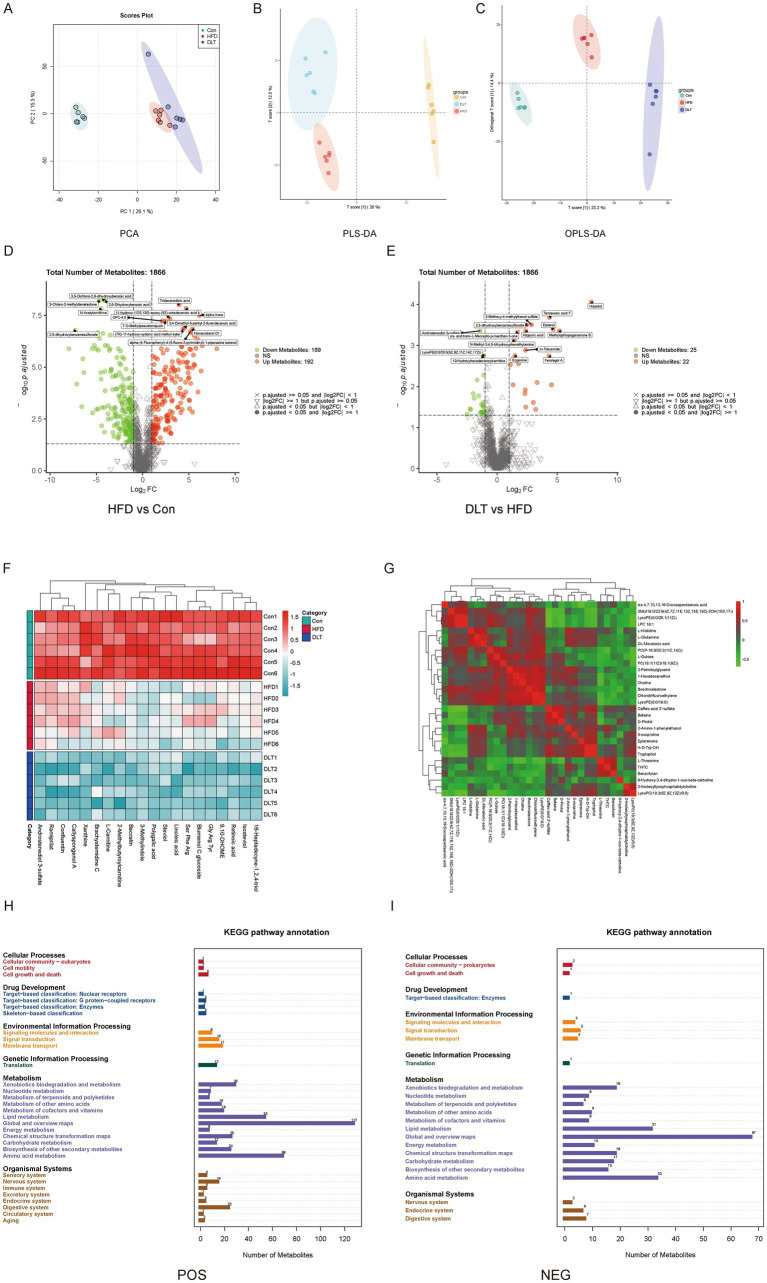
Untargeted serum metabolomics reveals the regulatory effects of DLT on metabolic pathways in HFD-fed mice. **(A)** Principal component analysis (PCA) score plot. **(B)** Partial least squares-discriminant analysis (PLS-DA) score plot. **(C)** Orthogonal projections to latent structures-discriminant analysis (OPLS-DA) score plot. **(D)** Volcano plot of differentially expressed metabolites (DEMs) between the HFD and Control groups. Red circles indicate significantly up-regulated metabolites (log₂FC >1, *p*-adj <0.05), green circles indicate down-regulated metabolites (log₂FC < 1, *p*-adj <0.05), and gray triangles represent non-significant metabolites. **(E)** Volcano plot of DEMs between the DLT and HFD groups. **(F)** Hierarchical clustering heatmap of the top 50 metabolites across experimental groups. Rows represent metabolites, columns represent groups. **(G)** Correlation heatmap of key serum metabolites. **(H)** KEGG pathway enrichment analysis of DEMs between the HFD and Control groups. **(I)** KEGG pathway enrichment analysis of DEMs between the DLT and HFD groups. The colored bars in **(H,I)** represent the major KEGG pathway categories: metabolism (purple), genetic information processing (green), environmental information processing (yellow), cellular processes (red), organismal systems (brown), and drug development (blue). The length of the bar represents the number of enriched metabolites in that pathway.

To uncover the systemic metabolic changes underpinning the observed therapeutic effects, we performed an untargeted metabolomic analysis of serum samples. Multivariate statistical analysis was employed to assess the overall metabolic shifts between the Con, HFD, and DLT groups. PCA showed a clear separation between the groups, with the first two principal components accounting for 41.4% of the variance (Figure 7A). This indicates that the diets and DLT treatment fundamentally altered the serum metabolome. This finding was corroborated by supervised methods, PLS-DA and OPLS-DA, which further confirmed the distinct metabolic profiles and the robustness of the group segregation (Figures 7B,C). These multivariate analyses confirm that DLT treatment induces a global metabolic shift that is distinct from both the healthy and HFD-diseased states.

To pinpoint the specific metabolites responsible for these shifts, we performed differential analysis. A total of 1866 metabolites were identified. Comparing the HFD group to the Con group revealed a substantial metabolic disruption, with 381 differentially expressed metabolites (DEMs) identified (192 up-regulated, 189 down-regulated) (Figure 7D). Strikingly, DLT intervention reversed many of these changes; when comparing the DLT group to the HFD group, only 47 DEMs were identified, suggesting a normalization of the metabolic profile (Figure 7E). These findings highlight the potent homeostatic effect of DLT, which appears to largely counteract the widespread metabolic disruption caused by a high-fat diet.

Hierarchical clustering analysis provided a visual confirmation of this restorative effect. The heatmap of metabolite profiles revealed that the DLT group’s metabolic pattern was more similar to the Con group than to the HFD group (Figure 7F). Specifically, DLT treatment effectively reversed HFD-induced alterations in key lipid metabolites, including retinoic acid, linoleic acid, and its derivatives. Furthermore, DLT intervention upregulated biomarkers of fatty acid β-oxidation, such as 2-methylbutyroylcarnitine and L-carnitine. The correlation heatmap revealed complex, coordinated relationships among metabolites, such as the strong positive correlation between phospholipids and the linkage between amino acids like L-histidine and L-glutamine, indicating a systemic re-wiring of metabolic networks (Figure 7G). These results demonstrate that DLT’s primary therapeutic mechanism involves restoring the balance of lipid and amino acid metabolism.

Finally, to place these metabolic changes into a functional context, we performed KEGG pathway enrichment analysis. In the HFD vs. Con comparison, the differentially abundant metabolites were primarily enriched in pathways related to amino acid, carbohydrate, and lipid metabolism (Figure 7H). In contrast, the DLT vs. HFD comparison revealed that DLT’s regulatory effects extended across multiple biological systems, including the nervous, endocrine, digestive, and immune systems (Figure 7I). This pathway analysis demonstrates that DLT is a multi-target therapeutic agent that modulates not only core metabolic pathways but also the broader physiological systems that are dysregulated in obesity.

### DLT modulates key serum metabolites in HFD-fed mice

**Figure 8:**
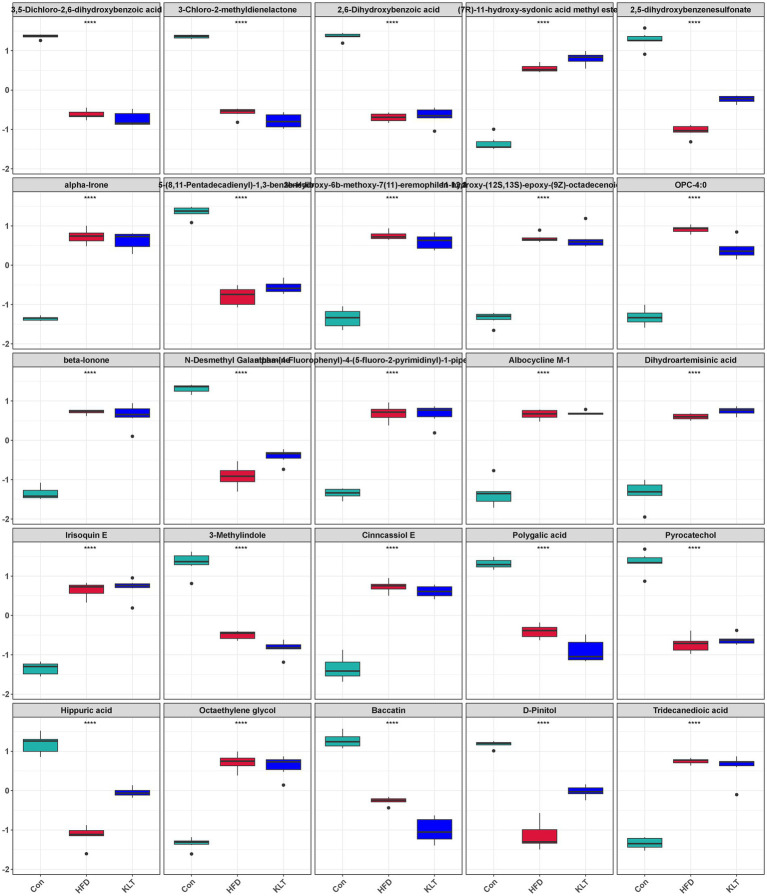
The relative abundance of key serum metabolites. The box plots display the top 25 differential metabolites identified in the study, including hippuric acid, beta-ionone, dihydroartemisinic acid, D-pinitol, and tridecanedioic acid, among others. Box plots represent the median, interquartile range, and outliers for each group. Statistical significance was determined by one-way ANOVA with Dunnett’s *post-hoc* test. ^***^p < 0.001 vs. the Con group; ^*^p < 0.05, ^**^p < 0.01 vs. the HFD group.

To identify the specific serum metabolites most impacted by HFD and DLT, we conducted a targeted analysis of the differential metabolites identified in our untargeted study. Box plot analysis revealed distinct expression patterns for numerous metabolites across the Con, HFD, and DLT groups (Figure 8). The HFD-induced metabolic disturbance was characterized by the significant upregulation of several key metabolites compared to the Con group, including hippuric acid, beta-ionone, and dihydroartemisinic acid. These specific metabolites may serve as potential biomarkers or contributors to HFD-induced physiological disorders. This finding confirms that HFD triggers a dysregulated metabolic signature for specific molecular compounds.

Crucially, DLT intervention demonstrated a potent restorative effect on these dysregulated metabolites. Treatment with DLT effectively reversed the HFD-induced changes, bringing the levels of several key metabolites back towards the normal ranges observed in the Con group. This normalization was particularly significant for hippuric acid, a metabolite linked to gut microbial metabolism of aromatic compounds. Similar beneficial effects were also observed for other important metabolites, including D-pinitol (an insulin-sensitizing compound) and tridecanedioic acid. These results demonstrate that DLT’s therapeutic effect is closely associated with the direct modulation of the serum abundance of specific, pathologically relevant metabolites.

### DLT reverses hepatic transcriptomic changes in HFD-fed mice

**Figure 9:**
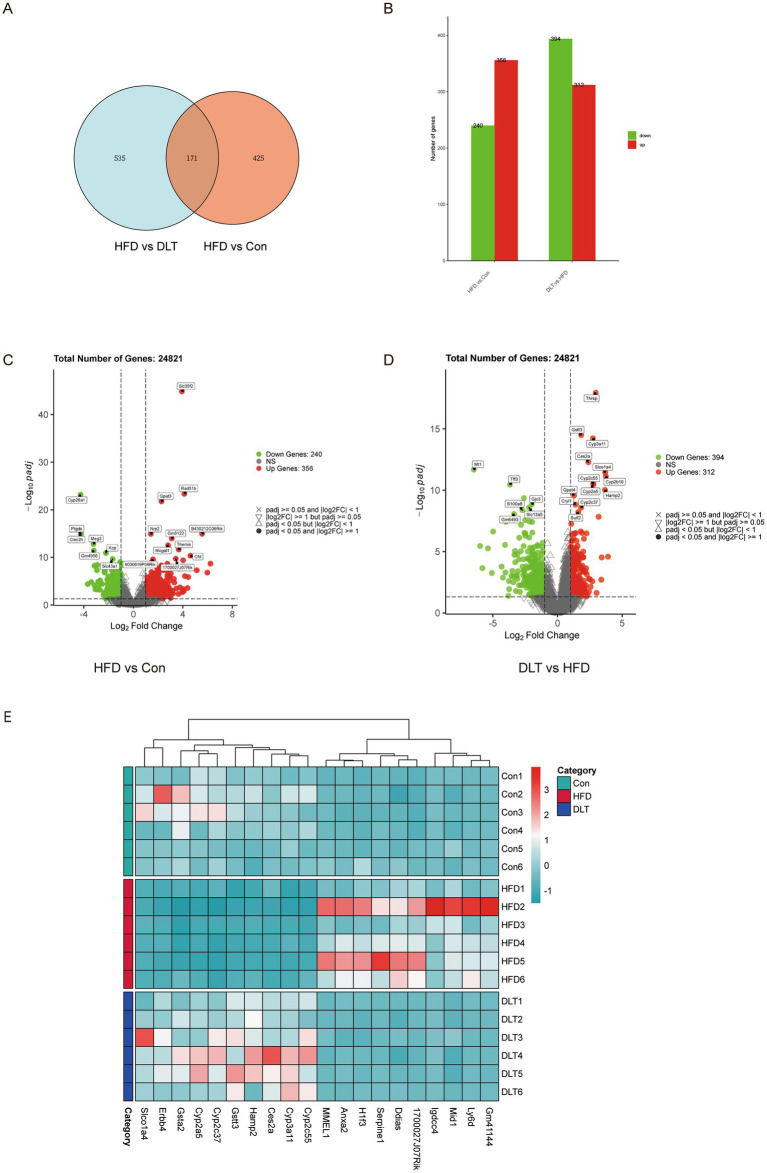
DLT treatment reverses the HFD-induced hepatic transcriptomic changes. **(A)** Venn diagram illustrating the number of differentially expressed genes (DEGs) in the HFD vs. Control (Con) and DLT vs. HFD comparisons, with the number of overlapping genes shown in the center. **(B)** Bar plot showing the total number of DEGs (upregulated and downregulated) for each comparison. **(C)** Volcano plot of DEGs between the HFD and Con groups. Red circles represent significantly up-regulated genes (log₂FC >0, *p*-adj <0.05) and blue circles represent significantly down-regulated genes (log₂FC <0, *p*-adj <0.05). **(D)** Volcano plot of DEGs between the DLT and HFD groups, with the same color scheme as **(C)**. **(E)** Heatmap of the top 50 DEGs across all individual samples (*n* = 6 per group). Rows represent genes, columns represent individual mouse samples, and the color gradient from cyan to red indicates the relative expression level. The hierarchical clustering dendrogram on the left shows the relationships between genes, and the dendrogram on top shows the relationships between samples, revealing distinct clustering of the treatment groups.

Having established a profound remodeling of the gut microbiome and serum metabolome, we next investigated the downstream effects on hepatic gene expression through RNA sequencing. HFD feeding induced substantial transcriptomic perturbations in the mouse liver. Comparative analysis identified 596 differentially expressed genes (DEGs) between the HFD and Con groups (356 upregulated, 240 downregulated) (Figures 9A–C). This confirms that HFD causes a significant reprogramming of hepatic function at the transcriptional level.

Crucially, DLT intervention was highly effective at counteracting these HFD-induced changes. A comparison of the DLT and HFD groups revealed 706 DEGs (312 upregulated, 394 downregulated) (Figures 9A,B,D). Importantly, of the 596 genes altered by HFD, 171 were significantly reversed by DLT treatment. This restorative effect was particularly evident for key metabolic pathways; while HFD upregulated pro-inflammatory mediators like *S100a8* and downregulated xenobiotic metabolism genes such as *Cyp3a11* and *Cyp2c55*, DLT treatment effectively normalized their expression. DLT further promoted the upregulation of beneficial detoxifying enzymes (e.g., *Ces2a*, *Cyp2a5*) and modulated the expression of key metabolic genes like the iron-regulatory hormone *Hamp2* (Figures 9C,D). These findings demonstrate that DLT directly reprograms the hepatic transcriptome, reversing the pathological gene expression signature induced by a high-fat diet.

Unsupervised hierarchical clustering provided a global confirmation of this restorative effect. The heatmap of DEGs revealed that the liver transcriptome of DLT-treated mice clustered closely with the healthy control group and was distinctly separated from the HFD group (Figure 9E). In all, hepatic transcriptome analysis reveals that DLT treatment reverses the HFD-induced gene expression signature, effectively reprogramming liver function towards a healthy state by suppressing inflammatory genes and restoring key metabolic and detoxification pathways.

### DLT targets metabolic and inflammatory pathways in HFD-induced steatosis

**Figure 10:**
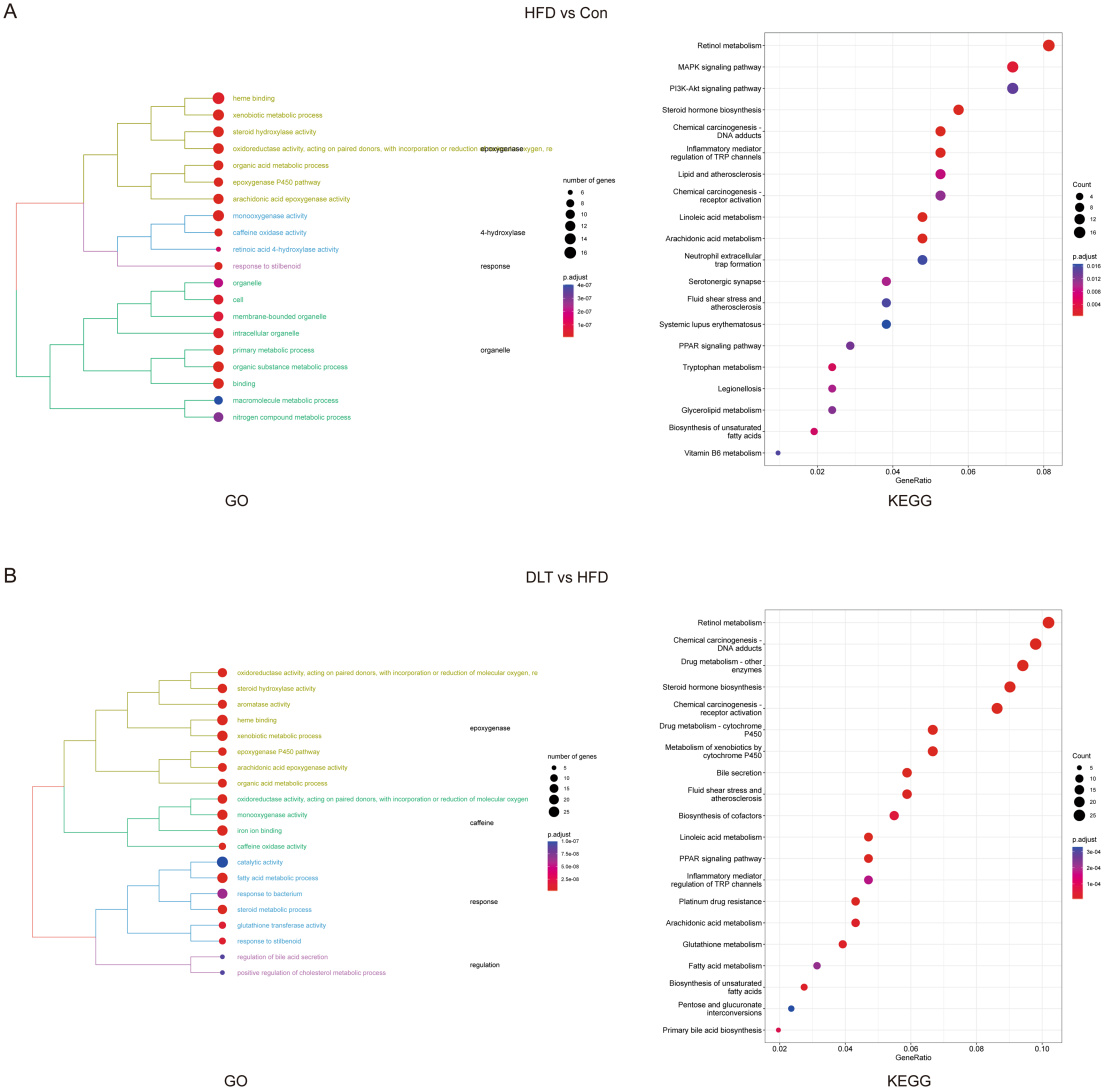
Functional enrichment analysis of differentially expressed genes (DEGs) in liver tissue. **(A)** Gene Ontology (GO) and Kyoto Encyclopedia of Genes and Genomes (KEGG) pathway enrichment analysis of DEGs between the HFD and Control groups. The bar chart shows the rich factor (ratio of the number of DEGs in a pathway to the total number of genes in that pathway) and the adjusted *p*-value (−log10) for each term. **(B)** GO and KEGG pathway enrichment analysis of DEGs between the DLT and HFD groups. The top 15 most significantly enriched terms (adjusted *p*-value <0.05) are displayed for each comparison.

To translate the lists of differentially expressed genes into biological pathways, we performed Gene Ontology (GO) and KEGG enrichment analyses. In the livers of HFD-fed mice, GO and KEGG analysis of DEGs revealed significant enrichment in biological processes that drive disease. This included the activation of key metabolic pathways like PPAR signaling and fatty acid degradation, alongside a strong activation of pro-inflammatory pathways, such as the TNF and NF-κB signaling cascades (Figure 10A). This pathway analysis confirms that HFD induces a combined state of metabolic dysfunction and chronic inflammation in the liver.

Following DLT intervention, the enrichment analysis revealed a powerful reversal of these HFD-induced pathways. GO terms related to antioxidant activity, mitochondrial function, and the negative regulation of inflammation were significantly enriched in the DLT vs. HFD comparison. Correspondingly, KEGG analysis showed that DLT treatment effectively upregulated pathways central to energy homeostasis, including oxidative phosphorylation and glutathione metabolism, while simultaneously downregulating pathways associated with inflammation and lipid accumulation (Figure 10B). These functional findings provide a molecular explanation for DLT’s therapeutic effects, demonstrating that it simultaneously enhances hepatic antioxidant capacity and corrects core metabolic signaling while directly suppressing inflammatory pathways.

### Integrated multi-omics analysis reveals DLT-mediated restoration of gut-liver axis homeostasis

**Figure 11:**
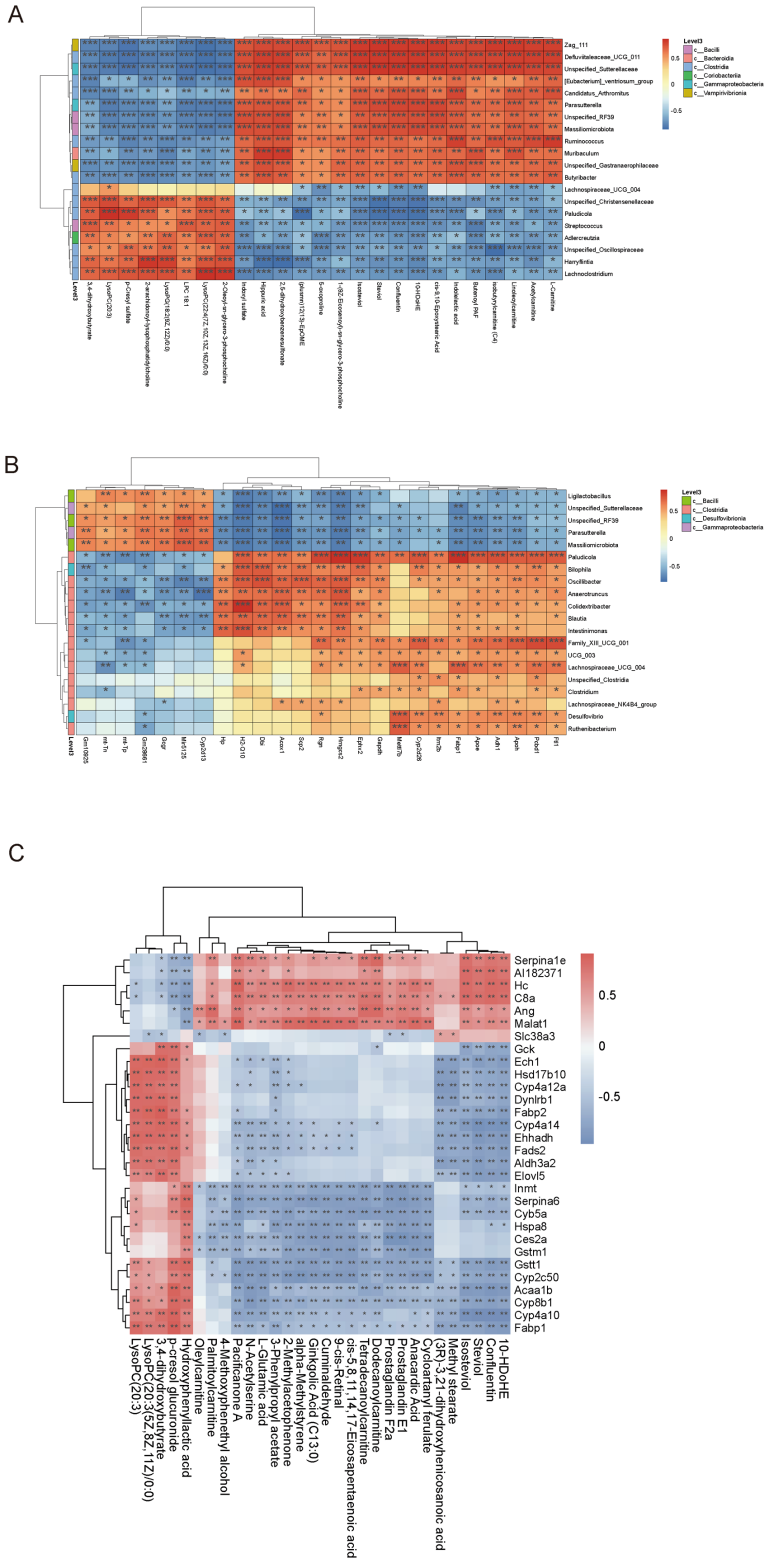
Integrated multi-omics correlation analysis reveals the DLT-mediated gut-liver axis mechanism. **(A)** Correlation maps between differentially abundant gut microbiota and differentially expressed serum metabolites. **(B)** Correlation maps between differentially abundant gut microbiota and differentially expressed hepatic genes. **(C)** Correlation maps between differentially expressed hepatic genes and differentially expressed serum metabolites.

To construct a comprehensive, systems-level model of DLT’s therapeutic action, we integrated our datasets from gut microbiota, liver transcriptomics, and host metabolomics. This multi-omics approach revealed a coherent regulatory network that correlates with the gut-liver axis, connecting the gut, liver, and systemic metabolism. The first layer of integration, correlating gut microbiota with circulating metabolites, revealed stark contrasts between the groups. We observed that HFD-enriched bacteria, particularly *Ruminococcus*, showed strong positive correlations with harmful gut-derived metabolites like p-Cresyl sulfate and Indoxyl sulfate, which are known uremic toxins that drive systemic inflammation (Figure 11A). This suggests a potential link between HFD-induced dysbiosis and the production of harmful systemic metabolites. In contrast, DLT treatment promoted beneficial bacteria such as *Lachnospiraceae_UCG_004*, which were positively correlated with anti-inflammatory lipids, including 10-HDoHE, a specialized pro-resolving mediator. This shift indicates that DLT remodels the gut to enhance the production of beneficial compounds, which is indicative of a regulatory relationship with systemic metabolic and inflammatory status.

To directly link these microbial shifts to hepatic responses, we correlated microbial abundance with liver gene expression (Figure 11B). This analysis revealed that HFD-associated pathobionts, like *Bilophila*, were positively correlated with hepatic expression of genes promoting steatosis, such as *Apoe* and *Fabp1*. Conversely, DLT-modulated bacteria were associated with the upregulation of hepatic xenobiotic metabolism genes, including *Cyp2d26*. These correlations provide compelling molecular evidence for a potential link that the reprogrammed gut microbiota is closely associated with the reshaping of hepatic transcriptional landscape.

The final layer integrated the liver transcriptome with the host metabolome to complete the regulatory circuit (Figure 11C). We found that in the DLT group, the upregulation of key hepatic genes involved in lipid catabolism and detoxification—including *Ces2a*, *Cyp4a10*, and *Fabp1*—was tightly coupled with the normalization of key metabolites involved in fatty acid transport and oxidation, such as Tetradecanoylcarnitine and Palmitoylcarnitine. This coordinated gene-metabolite network demonstrates that DLT orchestrates a hepatic transcriptional program that is closely associated with the normalization of systemic lipid metabolism.

This integrated multi-omics analysis delineates a potential “gut microbiota-liver transcriptome-host metabolome” regulatory network associated with the gut-liver axis, and all observed associations between microbial remodeling, metabolic normalization, and hepatic transcriptional reprogramming are based on correlative analyses. These findings provide a compelling exploratory framework for the gut-liver axis as a potential core pathway of DLT’s action, while direct causal validation requires further functional experiments.

## Discussion

In this study, we provide a comprehensive, multi-omics-driven mechanistic evaluation of Danlou tablet (DLT), demonstrating its potent efficacy against diet-induced obesity and insulin resistance. Our integrated multi-omics analysis uncovers a robust correlative pattern that DLT’s therapeutic effects are closely linked to the coordinated modulation of the gut-liver axis, a potential regulatory pathway where DLT systematically remodels the gut microbiota, which in turn is closely associated with the normalization ofhost serum metabolites and the reprogramming of hepatic gene expression that contributes to the restoration of metabolic homeostasis. These findings position the gut-liver axis as a potential core regulatory conduit for DLT’s action, supported by correlative multi-omics evidence and provide a robust scientific framework for its clinical translation in metabolic diseases. The potential modulation of the gut-liver axis by DLT is supported by multi-dimensional correlative evidence from our study: (1) DLT-induced gut microbial remodeling (e.g., reduced Firmicutes/Bacteroidota ratio, enriched SCFA-producing genera) is closely correlated with the normalization of serum metabolic profiles (e.g., enhanced fatty acid β-oxidation, reduced pro-inflammatory metabolites such as LPS); (2) DLT-modulated beneficial microbial genera are negatively correlated with hepatic pro-inflammatory gene expression and positively correlated with the upregulation of hepatic lipid catabolism and antioxidant genes; (3) the normalization of serum metabolites related to fatty acid metabolism (e.g., acylcarnitines) is tightly coupled with the transcriptional reprogramming of hepatic fatty acid oxidation pathways. These multi-omics correlations collectively construct a plausible exploratory model of the gut-liver axis as a potential regulatory pathway for DLT, and future functional experiments are essential to confirm the causality of this model and clarify the key molecular mediators of the gut-liver crosstalk.

The primary driver of DLT’s efficacy is closely associated with its profound impact on the gut microbial ecosystem. We observed that DLT treatment reversed the elevated Firmicutes/Bacteroidota (F/B) ratio, a hallmark of dysbiosis strongly associated with obesity (Magne et al., 2020). This phylum-level shift is critical, as an increased F/B ratio enhances energy harvest capacity, promoting fat storage and weight gain (Turnbaugh et al., 2006). Beyond this broad correction, DLT selectively suppressed pro-inflammatory-associated genera implicated in intestinal barrier damage in previous studies, such as *Mucispirillum* (Berry et al., 2015), while simultaneously enriching microbial genera that have been widely reported to be responsible for SCFA production in existing literature, including *Blautia*, *Roseburia*, and *Lactobacillus*. The enrichment of these genera, which are suggestive of enhanced SCFA production potential, is particularly noteworthy, as SCFAs are critical signaling molecules that enhance insulin sensitivity and exert anti-inflammatory effects (Pham et al., 2024; Xie et al., 2025). It should be emphasized that this inference about SCFA production is based on the reported functional characteristics of these genera in previous studies rather than direct functional evidence from the current 16S rRNA sequencing data, which cannot resolve species/strain-level functional heterogeneity within the same genus. By reshaping the microbial community toward a profile that favors gut barrier integrity and is associated with the potential production of beneficial metabolites based on genus-level changes, DLT effectively targets the foundational pathologies of metabolic syndrome, which is indicative of a potential regulatory relationship with the gut-liver axis and systemic metabolic homeostasis.

This targeted microbial remodeling is closely associated with a systemic correction of metabolic dysregulation. A critical consequence of HFD-induced dysbiosis is metabolic endotoxemia, the translocation of bacterial lipopolysaccharide (LPS) into circulation, triggering the low-grade systemic inflammation that is a key driver of insulin resistance (Cani and Van Hul, 2024; Mohammad and Thiemermann, 2021; Mazaheri-Tehrani et al., 2025). Our findings show that DLT significantly reduced serum LPS, IL-1α, and IL-6 levels, indicating a potent anti-inflammatory effect likely stemming from improved gut barrier function. Concurrently, our metabolomics analysis revealed that DLT normalized the HFD-disrupted serum metabolic profile, most notably by enhancing fatty acid β-oxidation. The upregulation of L-carnitine and acylcarnitines in DLT-treated mice points to increased mitochondrial fatty acid transport and oxidation, a mechanism known to alleviate the lipotoxicity that drives insulin resistance (Virmani and Cirulli, 2022; Marcovina et al., 2013). Thus, DLT breaks the vicious cycle where incomplete fatty acid oxidation leads to the accumulation of toxic lipid intermediates that impair insulin signaling (Mihalik et al., 2010).

Complementing the systemic regulation associated with the gut-liver axis, these blood-entering components may serve as potential candidate active constituents involved in DLT’s regulation of obesity and insulin resistance. Serum pharmacochemical analysis identified 33 blood-absorbed components in DLT-treated mice, including 27 prototypes and 6 metabolites (Supplementary Table S2). These absorbed constituents span diverse chemical classes, reflecting the complexity of the DLT formula: isoflavonoids (e.g., puerarin, 3′-hydroxypuerarin, and calycosin-7-O-β-D-glucoside); phenolic acids (e.g., ferulic acid); terpene glycosides (e.g., paeoniflorin); steroidal saponins (e.g., astragaloside IV and soyasaponin Bb); diterpenoids (e.g., tanshinone I, tanshinone IIA, and neocryptotanshinone); and triterpenoids (e.g., alisol A and alisol B). Among the prototype compounds, puerarin and its derivatives from *Pueraria lobata* are key isoflavonoids that have been widely reported to improve insulin sensitivity and regulate lipid metabolism by activating the AMPK signaling pathway (Xu et al., 2021; Gong et al., 2024; Liao et al., 2026). Paeoniflorin from *Paeonia lactiflora* Pall. and astragaloside IV from *Astragalus membranaceus* also exert synergistic effects: paeoniflorin alleviates hepatic inflammation, while astragaloside IV enhances mitochondrial fatty acid oxidation in the liver—mirroring the multi-component, multi-target characteristics of TCM observed in previous studies (Liu et al., 2026; Guo et al., 2025; Luo et al., 2021). Additionally, tanshinone I and tanshinone IIA from *Salvia miltiorrhiza* Bge. contribute to DLT’s anti-oxidative and anti-inflammatory effects by scavenging reactive oxygen species and inhibiting the NF-κB pathway, which aligns with the reported roles of liposoluble components in *Salvia miltiorrhiza*. Furthermore, the identification of six metabolites—resulting from Phase I biotransformations such as hydroxylation (affecting compounds like dihydrotanshinone I and pekinenal) and demethylation (affecting neocryptotanshinone and taiwaniaquinone A)—indicates that some TCM components require pharmacological activation to exert therapeutic effects. These reported biological activities of individual components suggest that the absorbed serum components of DLT may synergistically participate in its regulatory effects on metabolic syndrome, but the direct evidence for their role as key active constituents mediating microbial remodeling and metabolic improvement is still lacking, and the specific functional contributions of each component and their synergistic mechanisms need to be further verified by targeted experiments such as component knockout, activity evaluation and molecular docking.

Our integrated multi-omics analysis provides compelling correlative evidence that these gut and systemic effects converge on the liver. Correlation analysis established a clear link between DLT-modulated microbiota and the reprogramming of the hepatic transcriptome. For instance, HFD-associated bacteria like *Bilophila* correlated with hepatic lipid accumulation genes, whereas DLT-enriched taxa correlated with detoxification pathways. This gut-liver crosstalk is supported by our transcriptomic data, which showed that DLT treatment upregulated hepatic genes involved in the PPAR signaling pathway and glutathione metabolism. The activation of PPARs is a cornerstone of metabolic regulation, promoting fatty acid oxidation and improving insulin sensitivity (Vargas et al., 2011), while enhanced glutathione metabolism bolsters the liver’s antioxidant defenses (Zhou et al., 2025). The tight correlation between these upregulated hepatic genes and the normalized serum acylcarnitine profile confirms that DLT activates hepatic fatty acid β-oxidation, providing a clear molecular basis for its lipid-lowering and insulin-sensitizing effects that are closely associated with gut-liver axis modulation.

This study has several limitations that must be acknowledged. First, while our integrated multi-omics approach provides robust correlative evidence for the involvement of the gut-liver axis in DLT’s therapeutic effects, this study lacks direct functional validation experiments to establish a causal relationship. Future studies will employ classic microbiome functional verification strategies, including: (1) fecal microbiota transplantation (FMT) to transplant the gut microbiota of DLT-treated mice into germ-free or HFD-induced obese mice, to verify whether the beneficial metabolic effects of DLT can be transferred with the microbiota; (2) antibiotic depletion of the gut microbiota to confirm whether the therapeutic efficacy of DLT is attenuated or abrogated in microbiota-deficient mice; (3) targeted supplementation of DLT-enriched beneficial microbial genera (e.g., Lactobacillus, Blautia) or microbial metabolites (e.g., short-chain fatty acids, SCFAs) to validate their direct regulatory effects on hepatic metabolism and insulin sensitivity; and (4) pathway inhibition experiments (e.g., blocking key hepatic metabolic pathways or SCFA receptor signaling) to clarify the downstream molecular nodes of the gut-liver axis modulated by DLT. Second, the 16S rRNA sequencing employed in this study only achieves genus-level taxonomic resolution, which cannot resolve the functional heterogeneity of different species or strains within the same genus. Thus, the functional inferences (e.g., SCFA production, anti-inflammatory effects) linked to genus-level microbial abundance changes are only suggestive and supportive based on previous literature reports, rather than direct functional proof. Future studies will employ metagenomic sequencing to achieve species/strain-level resolution and conduct *in vitro* functional experiments (e.g., SCFA detection, co-culture experiments) to verify the actual metabolic and immunomodulatory functions of the DLT-modulated microbial taxa. Third, although we preliminarily identified 171 chemical components in DLT and 33 absorbed serum components, the current data are only a preliminary chemical profiling and lack direct experimental evidence to determine which specific compounds are the key active constituents responsible for microbial remodeling and metabolic improvement. Future studies should focus on bioactivity-guided fractionation, combined with *in vitro* cell experiments and *in vivo* animal models, to isolate and identify the key active constituents of DLT, and verify their specific regulatory effects and molecular targets on the gut microbiota, host metabolome and hepatic transcriptome. Finally, while our mouse model recapitulates many features of human metabolic syndrome, the findings ultimately require validation in human clinical trials.

In conclusion, our study demonstrates that DLT ameliorates obesity, insulin resistance, and related metabolic disorders by fundamentally remodeling the gut microbiota, and this remodeling is closely associated with the modulation of gut-liver axis-related metabolic and transcriptional networks, which contributes to the restoration of metabolic homeostasis. By integrating 16S rRNA sequencing, metabolomics, and transcriptomics, we have elucidated a holistic mechanism that aligns with the multi-component, multi-target nature of traditional Chinese medicine. The genus-level microbial analysis provides suggestive evidence for the potential functional changes of the gut microbiota induced by DLT, and these findings not only provide a strong scientific rationale for the clinical use of DLT in treating T2DM and related conditions, but also establish a critical exploratory framework for future studies to validate the causal role of the gut-liver axisand highlight the therapeutic potential of targeting the gut-liver axis to manage complex systemic diseases.

## Data Availability

The metabolomics data presented in the study are available in the MetaboLights repository, accession number MTBLS14750. The gut microbiota 16S rRNA sequencing data are available in the NCBI Sequence Read Archive (SRA), accession number PRJNA1445993. The liver transcriptomics RNA-seq data are available in the NCBI Sequence Read Archive (SRA), accession number PRJNA1449355.

## Glossary

ALT: Alanine aminotransferase
ANOVA: Analysis of variance
AST: Aspartate aminotransferase
AUC: Area under the curve
CAT: Catalase
cDNA: Complementary DNA
Con: Control
DEGs: Differentially expressed genes
DLT: Danlou tablet
DLT-H: High-dose Danlou tablet
DLT-L: Low-dose Danlou tablet
ELISA: Enzyme-linked immunosorbent assay
FBG: Fasting blood glucose
GSH-Px: Glutathione peroxidase
GTT: Glucose tolerance test
HDL-C: High-density lipoprotein cholesterol
HE: Hematoxylin and eosin
HFD: High-fat diet
HOMA-IR: Homeostasis model assessment of insulin resistance
IL-1β: Interleukin-1β
IL-6: Interleukin-6
IR: Insulin resistance
ITT: Insulin tolerance test
KEGG: Kyoto Encyclopedia of Genes and Genomes
LDL-C: Low-density lipoprotein cholesterol
LPS: Lipopolysaccharide
MDA: Malondialdehyde
MET: Metformin
OD: Optical density
PAS: Periodic acid–Schiff
PCA: Principal component analysis
PICRUSt: Phylogenetic Investigation of Communities by Reconstruction of Unobserved States
SCFA: Short-chain fatty acid
SEM: Standard error of the mean
SOD: Superoxide dismutase
T2DM: Type 2 diabetes mellitus
TC: Total cholesterol
TCM: Traditional Chinese medicine
TG: Triglycerides
UPLC-Q-TOF-MS: Ultra performance liquid chromatography-quadrupole-time of flight-mass spectrometry
