# Supplementary material for: Synergistic modulation of the gut microbiome-liver-host metabolome axis associates with the therapeutic efficacy of Danlou tablet against metabolic syndrome
Source: Front Microbiol. 2026 Jun 19;17:1808318. doi: 10.3389/fmicb.2026.1808318 (PMC13329814; doi:10.3389/fmicb.2026.1808318)
Supplement: Supplementary file 3 [file Supplementary_file_1.DOCX]

Supplementary Methods

**Contents**

S1. Animals, Diet, and Experimental Design

S2. Glucose Tolerance Test (GTT) and Insulin Tolerance Test (ITT)

S3. Serum Preparation, Biochemistry, ELISA, and Multiplex Immunoassay

S4. Tissue Collection, Organ Index, and Histological Staining (H&E, Oil Red O, PAS)

S5. 16S rRNA Gene Sequencing and Microbiome Analysis

S6. Untargeted Serum Metabolomics by UHPLC-Q-TOF-MS

S7. Liver Transcriptomic Profiling and Bioinformatic Analysis

S8. Expanded Statistical Methods

Supplementary Methods S1. Animals, Diet, and Experimental Design

Animals and housing. 6-week-old male C57BL/6N mice were obtained from Beijing Vital River Laboratory Animal Technology Co., Ltd. The mice were housed in a clean-grade animal facility, maintained at 22-24 °C with 58-60% humidity under a 12-hour light/dark cycle. Mice had ad libitum access to food and water.

Diets. The Control group was fed a standard chow diet (3.0 kcal/g; 25% protein, 60% carbohydrate, 15% fat). All other groups were fed a high-fat diet (5.24 kcal/g), rich in lard as the primary fat source, with a composition of 20% protein, 20% carbohydrate, and 60% fat.

Experimental design and interventions. After 1-week acclimation, mice were randomly assigned to five groups (n = 10 per group): control (Con, chow diet), high-fat diet (HFD), metformin (MET, 100 mg/kg), low-dose DLT (DLT-L, 20.83 g/kg), and high-dose DLT (DLT-H, 41.66 g/kg). MET, DLT-L, and DLT-H were administered once daily by oral gavage; Con and HFD received an equal volume of purified water. The intervention lasted 22 weeks. General health status was monitored daily, body weight was recorded weekly, and food intake was measured daily during the final week.

Ethics approval. Experimental protocols were approved by the Experimental Animal Ethics Committee of Henan University of Chinese Medicine (Approval No. IACUC-202403008).

Supplementary Methods S2. Glucose Tolerance Test (GTT) and Insulin Tolerance Test (ITT)

GTT and ITT were performed to assess glucose and insulin homeostasis. For GTT (week 20), mice were fasted for 16 h, weighed, and injected intraperitoneally with a 20% glucose solution at 2 g/kg body weight. Blood glucose was measured from the tail vein using a handheld glucometer at 0, 15, 30, 60, 90, and 120 min post-injection. For ITT (week 22), mice were fasted for 6 h, weighed, and injected intraperitoneally with insulin at 0.75 U/kg body weight, followed by blood glucose measurements at the same time points. Glucose/insulin tolerance curves were generated, and AUC was calculated using the trapezoidal method for quantitative comparison.

Supplementary Methods S3. Serum Preparation, Biochemistry, ELISA, and Multiplex Immunoassay

For serum biochemistry and cytokine analyses, mice were fasted for 12 h and anesthetized with intraperitoneal pentobarbital (40 mg/kg). Blood was collected from the orbital vein, allowed to clot at room temperature for 2 h, and centrifuged at 3000 rpm for 15 min at 4°C. Serum was aliquoted and stored at -20°C until analysis. Serum TC, TG, LDL-C, HDL-C, ALT, and AST were quantified using a Hitachi Chemistry Analyzer 7060. Serum LPS, CAT, SOD, MDA, and GSH-Px were measured using commercial ELISA kits according to the manufacturers’ instructions. Serum insulin, leptin, resistin, IL-1α, and IL-6 were determined using a multiplex immunoassay panel. HOMA-IR was calculated as: HOMA-IR = [fasting blood glucose (mmol/L) × fasting serum insulin (mU/L)] / 22.5. Reagent sources and suppliers are provided in Supplementary Table S1.

Supplementary Methods S4. Tissue Collection, Organ Index, and Histological Staining

After terminal blood collection, mice were euthanized. The liver, subcutaneous adipose tissue (SAT), and epididymal adipose tissue (EAT) were excised and weighed. Organ indices were calculated as: organ index (%) = tissue wet weight (g) / final body weight (g) × 100.

H&E staining. Liver and epididymal adipose tissues were fixed in 4% paraformaldehyde, paraffin-embedded, and sectioned at 5 μm. Sections were deparaffinized in xylene, rehydrated through graded ethanol, and stained with hematoxylin and eosin using routine procedures. After dehydration and clearing, sections were mounted and examined under light microscopy.

Oil Red O staining. For assessment of hepatic lipid accumulation, liver tissues were embedded in OCT compound, frozen, and sectioned at 8-10 μm. Sections were fixed in 4% paraformaldehyde for 30 min, rinsed, briefly equilibrated in 60% isopropanol, and stained with filtered Oil Red O working solution at room temperature for 15 min. Sections were differentiated in 60% isopropanol, counterstained with hematoxylin, blued under running water, and mounted with glycerin gelatin for microscopic evaluation.

PAS staining. Paraffin sections were deparaffinized, rehydrated, and rinsed. Sections were incubated with PAS oxidant for 5 min, stained with Schiff reagent for 10 min in the dark, and counterstained with hematoxylin. After bluing in 1×PBS, sections were dehydrated through graded ethanol, cleared in xylene, mounted with neutral gum, and imaged under light microscopy.

Supplementary Methods S5. 16S rRNA Gene Sequencing and Microbiome Analysis

Fecal sampling and DNA extraction. Fresh fecal pellets were collected from each mouse at week 20 and immediately stored at -80°C. Total microbial genomic DNA was extracted using the cetyltrimethylammonium bromide (CTAB) method. DNA quantity and integrity were assessed by 1% agarose gel electrophoresis, and DNA was diluted to 1 ng/μL with sterile water prior to amplification.

Amplicon PCR and library construction. The V3-V4 region of the bacterial 16S rRNA gene was amplified using barcoded primers 341F (5′-CCTAYGGGRBGCASCAG-3′) and 806R (5′-GGACTACNNGGGTATCTAAT-3′). PCR was performed in 15 μL reactions containing Phusion® High-Fidelity PCR Master Mix, 2 μM of each primer, and ~10 ng of template DNA. Cycling conditions were: 98°C for 1 min; 30 cycles of 98°C for 10 s, 50°C for 30 s, and 72°C for 30 s; followed by 72°C for 5 min. Amplicons were verified by 2% agarose gel electrophoresis, pooled at equimolar concentrations, and purified using a Universal DNA Purification Kit. Indexed sequencing libraries were prepared using the NEBNext® Ultra DNA Library Prep Kit for Illumina. Library quality was evaluated on an Agilent 5400 system, and libraries were sequenced on an Illumina platform using paired-end 2 × 250 bp reads.

Bioinformatic processing. Sequence data were processed in QIIME2 (v2019.1). Demultiplexed reads were quality-filtered, denoised, and chimera-checked using the DADA2 plugin to generate amplicon sequence variants (ASVs) and an ASV feature table. Taxonomy was assigned using the feature-classifier plugin against the Greengenes database (release 13_8; 99% OTUs; V3-V4 region).

Diversity and differential abundance analyses. Alpha diversity (observed ASVs (observed features), Chao1, Shannon, and Faith’s PD) and beta diversity (Bray-Curtis and weighted UniFrac) were calculated to characterize within- and between-sample community differences. Differential abundance was assessed using complementary statistical approaches to improve robustness, including ANCOM and LEfSe/DESeq2, with ANOVA-based testing applied where appropriate for group-level comparisons.

Functional inference. The functional potential of microbial communities was inferred using PICRUSt.

Supplementary Methods S6. Untargeted Serum Metabolomics by UHPLC-Q-TOF-MS

Sample extraction. Serum (100 μL) was extracted by adding 400 μL of ice-cold 80% methanol. Samples were vortexed, incubated on ice for 5 min, and centrifuged at 15,000 × g for 20 min at 4°C. The supernatant was diluted to a final methanol concentration of 53%, centrifuged again, and the resulting supernatant was used for LC-MS analysis.

UHPLC conditions. Separation was performed on a C18 column maintained at 40°C with a flow rate of 0.2 mL/min. Mobile phase A consisted of 0.1% formic acid in water and mobile phase B was methanol. The gradient program was: 2% B (0-1.5 min), 2-85% B (1.5-3 min), 85-100% B (3-10 min), 100-2% B (10-10.1 min), and 2% B (10.1-12 min).

Mass spectrometry acquisition. Data were acquired on a quadrupole time-of-flight (Q-TOF) mass spectrometer equipped with an electrospray ionization (ESI) source in both positive and negative ion modes over m/z 100-1500. Key parameters were: spray voltage 3.5 kV, sheath gas 35 psi, auxiliary gas 10 L/min, and ion transfer tube temperature 320°C. Data-dependent MS/MS acquisition was performed to support metabolite annotation.

Data preprocessing and feature annotation. Raw data files were converted to mzXML using ProteoWizard and processed using XCMS for peak detection, retention-time alignment, and quantification. Peak areas were normalized using a consistent reference-based approach (implemented as normalization to the first injected sample in the processing workflow) to facilitate between-sample comparability. Metabolites were annotated by MS/MS spectral matching against public databases (e.g., HMDB, LIPID MAPS, and KEGG) with a mass tolerance of 10 ppm. Features detected in blank samples were removed as background ions. The resulting metabolite feature matrix was used for downstream statistical and pathway analyses.

Supplementary Methods S7. Liver Transcriptomic Profiling and Bioinformatic Analysis

RNA extraction and quality control. Liver tissues were harvested, snap-frozen in liquid nitrogen, and stored at -80°C. Total RNA was extracted using TRIzol reagent according to the manufacturer’s protocol. RNA integrity and potential genomic DNA contamination were inspected by 1% agarose gel electrophoresis. RNA purity was assessed using NanoPhotometer, with acceptance criteria of OD260/280 ~2.0 and OD260/230 ≥2.0. RNA integrity was further evaluated on an Agilent 2100 Bioanalyzer using the RNA Nano 6000 Assay Kit, and samples with RIN ≥ 7.0 were used for library construction.

Library preparation and sequencing. Sequencing libraries were constructed from ≥1 μg total RNA using the NEBNext® Ultra™ RNA Library Prep Kit for Illumina. Poly(A)+ mRNA was enriched using oligo(dT) magnetic beads, fragmented, and reverse-transcribed to cDNA. Following end repair, adapter ligation, and size selection (250-300 bp inserts), libraries were treated with USER enzyme and PCR-amplified. Library concentration was assessed by Qubit, insert-size distribution by Agilent 2100 Bioanalyzer, and molarity by quantitative RT-PCR. Libraries were pooled and sequenced on an Illumina platform using 150 bp paired-end reads.

Read processing, alignment, and quantification. Raw FASTQ reads were processed using Trimmomatic to remove adapter sequences and low-quality bases. Clean reads were aligned to the mouse reference genome (GRCm39) using HISAT2 (default parameters). Transcript assembly and expression quantification (FPKM) were performed using StringTie.

Differential expression and functional interpretation. Differentially expressed genes (DEGs) were identified using DESeq2 with multiple-testing correction; genes with adjusted P < 0.05 were considered significant. Functional enrichment analyses for GO terms and KEGG pathways were conducted using clusterProfiler. Protein-protein interaction networks were explored using STRING.

Ancillary analyses. Alternative splicing events were analyzed using rMATS. SNP calling and annotation were performed using GATK and SnpEff, respectively.

Supplementary Methods S8. Expanded Statistical Methods

All data are expressed as mean ± SEM. Statistical analyses were performed using SPSS (version 25.0). Normality was assessed using the Shapiro-Wilk test. For comparisons among multiple groups, one-way ANOVA was applied; when an overall group effect was detected, post-hoc comparisons were performed using Dunnett’s C test. Longitudinal body-weight data were analyzed using two-way repeated-measures ANOVA followed by Bonferroni-adjusted post-hoc tests. A two-sided P < 0.05 was considered statistically significant.
