# Supplementary material for: Synergistic modulation of the gut microbiome-liver-host metabolome axis associates with the therapeutic efficacy of Danlou tablet against metabolic syndrome
Source: Front Microbiol. 2026 Jun 19;17:1808318. doi: 10.3389/fmicb.2026.1808318 (PMC13329814; doi:10.3389/fmicb.2026.1808318)
Supplement: Supplementary file 4 [file Supplementary_file_2.DOCX]

**Detailed Drugs and Reagents Information**

- **Pharmaceuticals**:
  - **Danlou Tablet (DLT)**: Provided by Jilin Connell Pharmaceutical Co., Ltd. (Jilin, China).
  - **Metformin**: Purchased from Beijing Jingfeng Pharmaceutical Group Co., Ltd. (Beijing, China).
- **Chemicals and Solutions**:
  - **Anhydrous Glucose**: Supplied by Guangzhou Guanghua Science and Technology Co., Ltd. (Guangzhou, China).
  - **Anhydrous Ethanol**: Supplied by Sangon Biotech (Shanghai) Co., Ltd. (Shanghai, China).
- **Histology Reagents**:
  - **Neutral Buffered Formalin**: Acquired from Tianjin Comio Chemical Reagent Co., Ltd. (Tianjin, China).
  - **Ethanol and Xylene**: Supplied by Tianjin Comio Chemical Reagent Co., Ltd. (Tianjin, China).
  - **Hematoxylin and Eosin (HE) Staining Kit**: Obtained from Beijing Solarbio Science & Technology Co., Ltd. (Beijing, China).
  - **Oil Red O Staining Kit**: Obtained from Beijing Solarbio Science & Technology Co., Ltd. (Beijing, China).
  - **Periodic Acid-Schiff (PAS) Staining Kit**: Obtained from Beijing Solarbio Science & Technology Co., Ltd. (Beijing, China).
- **Other Reagents**:
  - All other reagents were of analytical grade and purchased from **Sinopharm Chemical Reagent Co., Ltd. (Beijing, China)**.
- **Assay Kits**:
  - **Biochemical Assay Kits** for total cholesterol (TC), triglycerides (TG), low-density lipoprotein cholesterol (LDL-C), high-density lipoprotein cholesterol (HDL-C), alanine aminotransferase (ALT), and aspartate aminotransferase (AST) were provided by **Zhongsheng Beikong Bio-technology and Science, Inc.**
  - **Kits for Lipopolysaccharide (LPS)**, catalase (CAT), superoxide dismutase (SOD), malondialdehyde (MDA), and glutathione peroxidase (GSH-Px) were purchased from **Jiangsu Enzyme Immuno Technology Co., Ltd.**
  - **Multiplex Immunoassay Panel** for leptin, insulin, resistin, interleukin-1α (IL-1α), and interleukin-6 (IL-6) was supplied by **Merck KGaA (Germany)**.
